# Metabolomic profiles, polygenic risk scores and risk of rheumatoid arthritis: a population-based cohort study in the UK Biobank

**DOI:** 10.1136/rmdopen-2023-003560

**Published:** 2023-11-30

**Authors:** Xin-Yu Fang, Jie Zhang, Ting-Ting Qian, Peng Gao, Qing Wu, Quan Fang, Su-Su Ke, Rong-Gui Huang, Heng-Chuan Zhang, Ni-Ni Qiao, Yin-Guang Fan, Dong-Qing Ye

**Affiliations:** 1 Epidemiology and Biostatistics, School of Public Health, Anhui Medical University, Hefei, Anhui, China; 2 Inflammation and Immune Mediated Diseases Laboratory of Anhui Province, Hefei, Anhui, China; 3 School of Public Health, Anhui University of Science and Technology, Hefei, Anhui, China; 4 Anhui Institute of Occupational Safety and Health, Anhui University of Science and Technology, Hefei, China

**Keywords:** Epidemiology, Arthritis, Rheumatoid, Autoimmune Diseases

## Abstract

**Objective:**

To investigate the relationship between metabolomic profiles, genome-wide polygenic risk scores (PRSs) and risk of rheumatoid arthritis (RA).

**Methods:**

143 nuclear magnetic resonance-based plasma metabolic biomarkers were measured among 93 800 participants in the UK Biobank. The Cox regression model was used to assess the associations between these metabolic biomarkers and RA risk, and genetic correlation and Mendelian randomisation analyses were performed to reveal their causal relationships. Subsequently, a metabolic risk score (MRS) comprised of the weighted sum of 17 clinically validated metabolic markers was constructed. A PRS was derived by assigning weights to genetic variants that exhibited significant associations with RA at a genome-wide level.

**Results:**

A total of 620 incident RA cases were recorded during a median follow-up time of 8.2 years. We determined that 30 metabolic biomarkers were potentially associated with RA, while no further significant causal associations were found. Individuals in the top decile of MRS had an increased risk of RA (HR 3.52, 95% CI: 2.80 to 4.43) compared with those below the median of MRS. Further, significant gradient associations between MRS and RA risk were observed across genetic risk strata. Specifically, compared with the low genetic risk and favourable MRS group, the risk of incident RA in the high genetic risk and unfavourable MRS group has almost elevated by fivefold (HR 6.10, 95% CI: 4.06 to 9.14).

**Conclusion:**

Our findings suggested the metabolic profiles comprising multiple metabolic biomarkers contribute to capturing an elevated risk of RA, and the integration of genome-wide PRSs further improved risk stratification.

**WHAT IS ALREADY KNOWN ON THIS TOPIC**

- Limited research has been conducted on the relationship between circulating metabolomic profiles and the risk of rheumatoid arthritis (RA), and the existing studies have been relatively small in scale but have yielded promising results. Additionally, the literature lacks comprehensive assessments of the combined effects of metabolomic profiles and genetic predisposition on RA risk.

**WHAT THIS STUDY ADDS**

- This population-based prospective cohort study advances our knowledge by identifying 30 potential metabolites associated with future RA events, covering various molecular pathways including fatty acids, body fluid balance, glycolysis, inflammation and detailed lipoprotein particle composition. However, Mendelian randomisation analysis did not establish a causal link between these metabolites and RA.
- This study highlights the importance of a comprehensive metabolic profile in capturing potential RA risks and indicates a synergistic effect between high genetic susceptibility and unfavorable metabolic profiles in increasing the incidence of RA.

**HOW THIS STUDY MIGHT AFFECT RESEARCH, PRACTICE OR POLICY**

- Our findings suggest the identification of individuals who could benefit from precision interventions targeting specific metabolic pathways to prevent RA, especially those with high genetic predisposition. Understanding the combined effects of genetics and metabolomics could lead to more targeted and personalised approaches in RA prevention and management.

## Introduction

Rheumatoid arthritis (RA) is an autoimmune-based inflammatory disease featured by immune system cells invading the synovial tissue of the affected joint, causing progressive destruction of the affected joints and eventually leading to disability.^1^ It affects approximately 1% of the world’s population with significant socioeconomic consequences.^2^ To date, RA is still incurable and can be deadly due to severe complications, such as cardiovascular disease (CVD). Identifying individuals at the early stages of RA, that is, before overt clinical symptoms, has been challenging.^3^

Given that metabolites are involved in the body’s metabolic homeostasis and immune response,^4^ with the development of metabolomics technology in recent years,^5^ mining the potential of metabolites as disease-specific biomarkers has led to a research boom in RA.^6^ Through metabolomics, thousands of metabolites can be quantified in a rapid, simultaneous measurement, capturing specific biomarkers and revealing the consequences of genetic and environmental interactions.^7^ Currently, relevant studies on RA are still in scarcity. Previous research has brought some promising results^8–13^; however, the sample size and the coverage of metabolites are relatively small in these studies, and primarily cross-sectional and case–control studies, or do not take into account the heterogeneity of cases, which limits the extrapolation of research results, hindering their rapid translation into clinical applications. Furthermore, the potential benefits of combining metabolomics with genetics to identify individuals at the highest risk of RA remain largely unexplored.

Therefore, based on the recently available data in the UK Biobank, we delineated the potential relationship between nuclear magnetic resonance (NMR)-based circulating metabolic biomarkers and the risk of incident RA in the UK general population settings for the first time. In addition, genetic correlation and Mendelian randomisation analyses were designed to probe the possible causal relationships between metabolites and RA. Considering that genetic predisposition in combination with metabolomics may be helpful in risk prediction, we also constructed a genome-wide polygenic risk score (PRS) to explore the modification effect on metabolic profiles affecting the onset of RA.

## Methods

### Study population and data collection

UK Biobank (UKB) is a prospective cohort study established by the Medical Research Council and Wellcome Trust, which recruited ~500 000 participants aged 40–69 years across the UK during 2006–2010. These participants completed the survey at 1 of 22 assessment centres in England, Scotland and Wales, where they completed touchscreen and trained staff-led questionnaires, performed body measurements and provided biological samples, linked to health-related records and registries. Details of the UKB study design and population have been described previously.^14^

Of the initial 502 493 participants, 118 021 (23%) had completed metabolic biomarker profiling. Subsequently, 24 211 participants with prior RA and missing NMR biomarker (and follow-up) data were further excluded, leaving 93 800 (79.5%) in the primary analyses. Besides, in terms of studies involving genetic data, 21 397 participants who had missing genotypes or heterozygosity rate outliers, were sex inferred from the genotypes did not match their self-reported sex and were not of Caucasian ancestry or had relatives were removed, remaining 72 403 participants (online supplemental figure 1).

10.1136/rmdopen-2023-003560.supp1 Supplementary data

### Metabolic biomarker quantification

A high-throughput NMR-based metabolic biomarker platform was performed to measure the metabolomic profiling in the randomly selected EDTA plasma samples from the subset of the UKB participants. The measurements took place between June 2019 and April 2020, and 249 metabolic biomarkers (168 in absolute levels and 81 ratio measures) were simultaneously quantified (https://biobank.ndph.ox.ac.uk/ukb/ukb/docs/nmrm_companion_doc.pdf). These metabolic biomarkers cover cholesterol metabolism, fatty acid compositions, lipoprotein subclass distribution, and particle size and composition, as well as various low-molecular-weight metabolites, such as amino acids, ketones and glycolysis metabolites. Details of the NMR metabolomics experimentation have been described previously^15^ and epidemiological applications^16^ are recently being reviewed. In this study, we selected a subset of 143 metabolites (online supplemental table 1), which focused on those directly measured and those that cannot be inferred, for analysis.^17^ Currently, the UKB Nightingale NMR platform has received regulatory approvals, including the CE mark, and features 37 biomarkers covering the majority of metabolic pathways detected by NMR, which have been certified for broad diagnostic use.^18^ Moreover, these biomarker measurements are furnished in a format consistent with traditional clinical chemistry measurements, specifically absolute concentrations (https://research.nightingalehealth.com/clinically-validated-biomarkers).

### Ascertainment of RA cases

The self-report+related therapeutic drug use, and the hospital inpatient records (using international classification of disease 9th version (ICD-9), and international classification of disease (ICD) 10th version (ICD-10)), were combined to find out prior RA cases at the baseline. In detail, participants’ self-reported RA and records of those who had used steroids and the disease-modifying antirheumatic drugs for RA were obtained through a touchscreen questionnaire completed by participants and subsequently recorded by a trained staff. Hospital inpatient records are linked with National Health Service hospital inpatient data from Hospital Episode Statistics in England, the Scottish Morbidity Records and the Patient Episode Database for Wales, allowing for accurate identification of the first recorded date of each diagnosis. During the follow-up, incident RA cases in this study were identified from admission data using ICD-9 or ICD-10. Detailed information for codes used to identify RA cases in this study can be seen in online supplemental table 2.

### Genetic correlation and Mendelian randomisation analyses

To investigate the proportion of variance that metabolites and RA share due to genetic cause, we conducted the cross-trait linkage disequilibrium score regression (LDSC) to estimate genome-wide pairwise genetic correlation.^19^ Further, independent, genome-wide significant single nucleotide polymorphisms (SNPs) were used as instruments to perform the two-sample Mendelian randomisation (MR) analysis. We obtained the genome-wide association study (GWAS) summary statistics of candidate metabolites from the Integrative Epidemiology Unit (IEU) GWAS database (https://gwas.mrcieu.ac.uk; dataset ID: met-d). The GWAS summary statistics for RA were acquired from a European RA GWAS meta-analysis, encompassing 14 361 RA cases and 43 923 controls (http://plaza.umin.ac.jp/%7Eyokada/datasource/files/GWASMetaResults/RA_GWASmeta_European_v2.txt.gz). Besides, we also included the RA GWAS summary statistics from the FinnGen biobank analysis round 5 (6236 RA cases, 147 221 controls) for two-sample MR analysis. The relevant GWAS summary statistics were obtained from the IEU GWAS database (dataset ID: finn-b-M13_RHEUMA).

The pre-calculated linkage disequilibrium (LD) scores computed using 1000 Genomes European data provided by LDSC (https://data.broadinstitute.org/alkesgroup/LDSCORE/) were performed to calculate LD scores and genetic correlation. In performing the two-sample MR, we initially implemented a systematic approach to identify suitable SNPs as instrumental variables (IVs). The selection criteria encompassed the inclusion of SNPs demonstrating genome-wide significance (p<5×10^−8^) and the establishment of LD r^2^ cut-off at 0.001 during the clumping procedure. An in-depth description of the SNP selection methodology can be found in online supplemental file 1. Additionally, we assessed the F-statistics of the IVs, with all values exceeding 10, indicative of relatively low risk of weak instrument bias^20^ (online supplemental table 3). Then, we used the inverse-variance weighted method, MR-Egger method and weighted median method to pool the estimates from multiple SNPs.^21^

We also executed a one-sample MR analysis using imputed genotyping data (online supplemental text 1) from the UKB and genetic risk scores as instruments, which were based on selected SNPs and effect sizes derived from GWAS. A two-stage regression was conducted, initially predicting metabolite values through linear regression employing genetic instruments, followed by regressing RA on the predicted values using Cox regression. Both stages incorporated adjustments for covariates, including age, sex, the first 10 genetic principal components, genotyping batches and UKB assessment centres. The tests for weak instruments and statistical power can be found in online supplemental table 4. More detailed information on MR analyses can be seen in online supplemental text 2.

### Construction of PRS for RA

The PRS^22^ that captured an individual’s load of common genetic variants (variants in the major histocompatibility complex (MHC) locus, as well as non-MHC variants) associated with RA has been constructed to estimate the modification effects of genetic susceptibility. The PRS of RA combined PRS*_non-MHC_* and PRS*_HLA_*^23^ (online supplemental text 3). The PRS*_non-MHC_* included 58 SNPs associated with RA risk (online supplemental table 5), weighted by the natural logarithm of the published OR. PRS*_HLA_* for RA susceptibility incorporated three *HLA-DRB1* amino acid positions 11, 71 and 74 and one position 9 on each *HLA-B* and *HLA-DPB1*, weighted by published OR for each haplotype^24^ (online supplemental table 6). According to the distribution of PRS, individuals were categorised into low (tertile 1), intermediate (tertile 2) and high-grade (tertile 3) genetic risks of RA.

### Assessment of covariates

Covariates in the primary analyses included age (years), sex, assessment centre (22 centres), fasting duration (hours), educational level (university degree or other), smoking status (never, ever or current), body mass index (BMI, kg/m^2^), physical activity (summed metabolic equivalent task minutes per week for all activities). For female subgroup analyses, menopausal status and postmenopausal hormone use were additionally considered.^25 26^ During sensitivity analyses, the prevalent status of CVD, diabetes and cancer, as well as medication usage for lipid-lowering, non-steroidal anti-inflammatory drugs (NSAIDs) and traditional clinical blood measures (albumin (g/L), high-density lipoprotein (HDL)-cholesterol (mmol/L), low-density lipoprotein (LDL)-cholesterol (mmol/L), triglycerides (mmol/L) and C reactive protein (mg/L)) were adjusted accordingly. Traditional blood biochemistry biomarkers were assessed using the Beckman Coulter AU5800 system. Genetic analyses incorporated the first 10 genetic principal components (1–10) and genotyping batches (11 batches in the UK BiLEVE Axiom array and 95 batches in the Biobank Axiom array).

### Statistical analysis

Metabolite levels outside four IQRs from the median were considered outliers and excluded. All of the metabolic biomarkers in this study were scaled to SD units before analyses. Person-years of follow-up were defined from the date of initial recruitment to the incident RA, the date of death, loss of follow-up or end of follow-up (31 March 2017 for England; 31 October 2016 for Scotland and 31 January 2017 for Wales). A multivariable Cox proportional hazards model was applied to assess the association between all the 143 metabolic biomarkers and the risk of incident RA, with adjusting age, sex, assessment centres, fasting time, educational level, smoking status, BMI and physical activities. The assumption of proportional hazards was assessed by analysing the relationship between standardised Schoenfeld residuals and time, and no violation to the proportional hazards assumption was found. The false discovery rate (FDR)-corrected threshold was set to p<0.05. Then, these metabolic biomarkers (FDR p<0.05) were included as candidate metabolites in subsequent genetic analyses, where genetic correlation analyses, one-sample and two-sample MR were performed to further investigate the causal relationship between candidate metabolic biomarkers and RA.

To delineate comprehensive metabolic profiles for RA, we constructed a composite ‘metabolic risk score’ (MRS). Using a LASSO-Cox model, which uses L1 regularisation that adds a penalty equal to the absolute value of the coefficient magnitude for variable selection,^27^ we regressed RA risk on the set of 37 clinically validated metabolic biomarkers. A random subset of 60% of all study participants was selected as the training dataset and used fivefold cross-validation and minimised mean absolute error to optimise the regularisation parameter λ. The MRS was calculated as the weighted sum of the metabolic biomarkers with non-zero coefficients, where the weights were equal to the corresponding coefficients in the optimised LASSO-Cox model. The remaining study population was used as an internal validation dataset to validate the c-index of the MRS by comparison with the traditional risk factor combination (age, gender, smoking status, BMI, education), as well as to examine whether there was a significant time-varying effect on the MRS. Additionally, we used all 143 metabolites to construct an alternative MRS for sensitivity analysis, thereby assessing the robustness of the risk score formulation. The above process resulted in an MRS for main analyses comprised of the weighted sum of 17 biomarkers from the 37 clinically validated metabolic markers, and an alternative MRS for method validation comprised of the weighted sum of 6 biomarkers from all 143 NMR-quantified metabolites (weighted coefficients, see online supplemental tables 7 and 8). The performance of MRS in the test dataset and the examination of possible time-varying effects were presented in online supplemental figure 2.

The MRS was divided into four grades (0–50%, 50–75%, 75–90%, 90–100%) using percentiles, and the time resolution of cumulative incidence for each grade was further evaluated by the Kaplan-Meier curve. Then, the HR and 95% CI of RA risk according to MRS grades (0–50% as the reference level) were estimated using multivariate-adjusted Cox models that accounted for age, sex, assessment centres, fasting time, educational level, smoking status, BMI and physical activities. The Cox models were stratified by age, sex and rheumatoid factor and additionally adjusted prevalent diseases (CVD, cancer, diabetes), lipid-lowing drugs and NSAIDs or traditionally clinical blood measures (albumin, HDL-cholesterol, LDL-cholesterol, triglycerides, C reactive protein). The Spearman correlation coefficients between MRS and traditional clinical blood biochemistry measures were also checked and shown in online supplemental figure 3.

Sensitivity analyses were conducted to check the robustness and specificity of the study results. First, the incident RA events in the first 2 years from baseline were excluded to reduce the confounding of reverse causality. We also compared the effects of the MRS on the short-term (2–5 years) and long-term (>5 years) risk of RA to elucidate whether metabolite detection timing has an impact on predicting the risk of RA. Additionally, we tested whether MRS was associated with RA risk across different RA genetic risk subgroups. We classified participants into nine groups according to the pairwise combinations of MRS (favourable: 0–50%, moderate: 50–90% and unfavourable: 90–100%) and PRS of RA (low: tertile 1, medium: tertile 2 and high: tertile 3) to assess the combined effects of genetics and metabolics on the incidence of RA. The interaction effect between RA PRS and MRS was evaluated by adding a product term of PRS of RA and MRS in the Cox models.

Analyses were conducted using the LDSC software, and packages included ‘survival’, ‘glmnet’ and ‘TwoSampleMR’ in R (V.4.1.0) software. An FDR-corrected p<0.05 was considered statistically significant in association analyses of metabolomic biomarkers, and a two-sided p<0.05 was for other analyses.

## Results

At the baseline, 93 800 UKB participants with no prior RA and who completed metabolic profiling without missing data were included in this study. The average age (SD) of the participants was 59.7 (7.2) years, and most of them were women (64.4%). During the follow-up (median 8.2 years), 620 incident RA cases were identified according to the hospital registries recorded. Participants who developed RA were more likely to be former or current regular smokers, have higher levels of BMI, less physical activity time, and a higher prevalence of vascular or heart problems, cancer and diabetes than those who did not develop RA. They also had higher concentrations of C reactive protein and rheumatoid factors (table 1). A comparison of basic characteristics of participants with and without metabolic biomarker profiling was presented in online supplemental table 9. A flow diagram of eligible study participants and overall study design is shown in online supplemental figure 1.

**Table 1:** Population characteristics of the 620 incident RA events and 93 180 non-RA from UK Biobank in the current study

Characteristics	Incident RA		P value*
	Yes (n=620)	No (n=93 180)	
Age at blood collection (years)	59.7 (7.2)	56.8 (8.0)	<0.001
Time from blood collection to diagnosis	5.0 (2.3)		
Sex, n (%)			<0.001
Women	386 (62.3)	49 176 (52.8)	
Men	234 (37.7)	44 004 (47.2)	
Education level, n (%)			<0.001
University degree	117 (18.9)	28 891 (31.0)	
No university degree	503 (81.1)	64 289 (69.0)	
Smoking status, n (%)			<0.001
Never	274 (44.2)	50 813 (54.5)	
Previous	259 (41.8)	32 826 (35.2)	
Current	87 (14.0)	9541 (10.3)	
Physical activity (MET min/week)	2654.7 (2741.8)	2731.3 (3086.5)	<0.001
Body mass index (kg/m^2^)	29.2 (5.8)	25.5 (4.7)	<0.001
Fasting time (hours)	3.9 (2.6)	3.7 (2.4)	0.09
Genetic risk for RA†			<0.001
Low	121 (25.2)	24 171 (33.4)	
Medium	155 (32.3)	24 137 (33.3)	
High	204 (42.5)	24 095 (33.3)	
Clinical blood chemistry measures			
Total cholesterol (mmol/L)	5.66 (1.22)	5.72 (1.14)	0.21
HDL-cholesterol (mmol/L)	1.40 (0.38)	1.44 (0.37)	0.07
LDL-cholesterol (mmol/L)	3.54 (0.90)	3.59 (0.87)	0.19
Triglycerides (mmol/L)	1.85 (0.99)	1.78 (1.01)	0.08
Albumin (g/L)	44.22 (2.74)	45.24 (2.61)	<0.001
C reactive protein (mg/L)	5.34 (8.24)	2.56 (4.24)	<0.001
Rheumatoid factor‡ (IU/mL)	34.84 (27.33)	23.80 (19.24)	<0.001
Prevalent diseases, n (%)			
CVD	273 (44.0)	27 688 (29.7)	<0.001
Diabetes	52 (8.4)	4502 (4.8)	<0.001
Cancers	58 (9.4)	7196 (7.7)	0.15

*Quantitative and qualitative variables were expressed as means (SD) or number (percentages), and were compared using Student’s t-test or the χ^2^ test according to RA status, respectively.

†After genetic QC, there remained 480 incident RA and 72 403 non-RA cases in the genetic analysis. Genetic risk (low: tertile first of PRS, medium: tertile second of PRS, high: tertile third of PRS).

‡The number of missing values in incident RA groups was 497 (81.5%) and in non-RA groups was 85 556 (91.8%). The reason for missing rheumatoid factor measurements was due to the original value above or below the reportable limits, minorly unrecoverable aliquot problems or no returned data.

CVD, cardiovascular disease; HDL, high-density lipoprotein; LDL, low-density lipoprotein; MET, metabolic equivalent task; PRS, polygenic risk score; QC, quality control; RA, rheumatoid arthritis.

**Figure 1:**
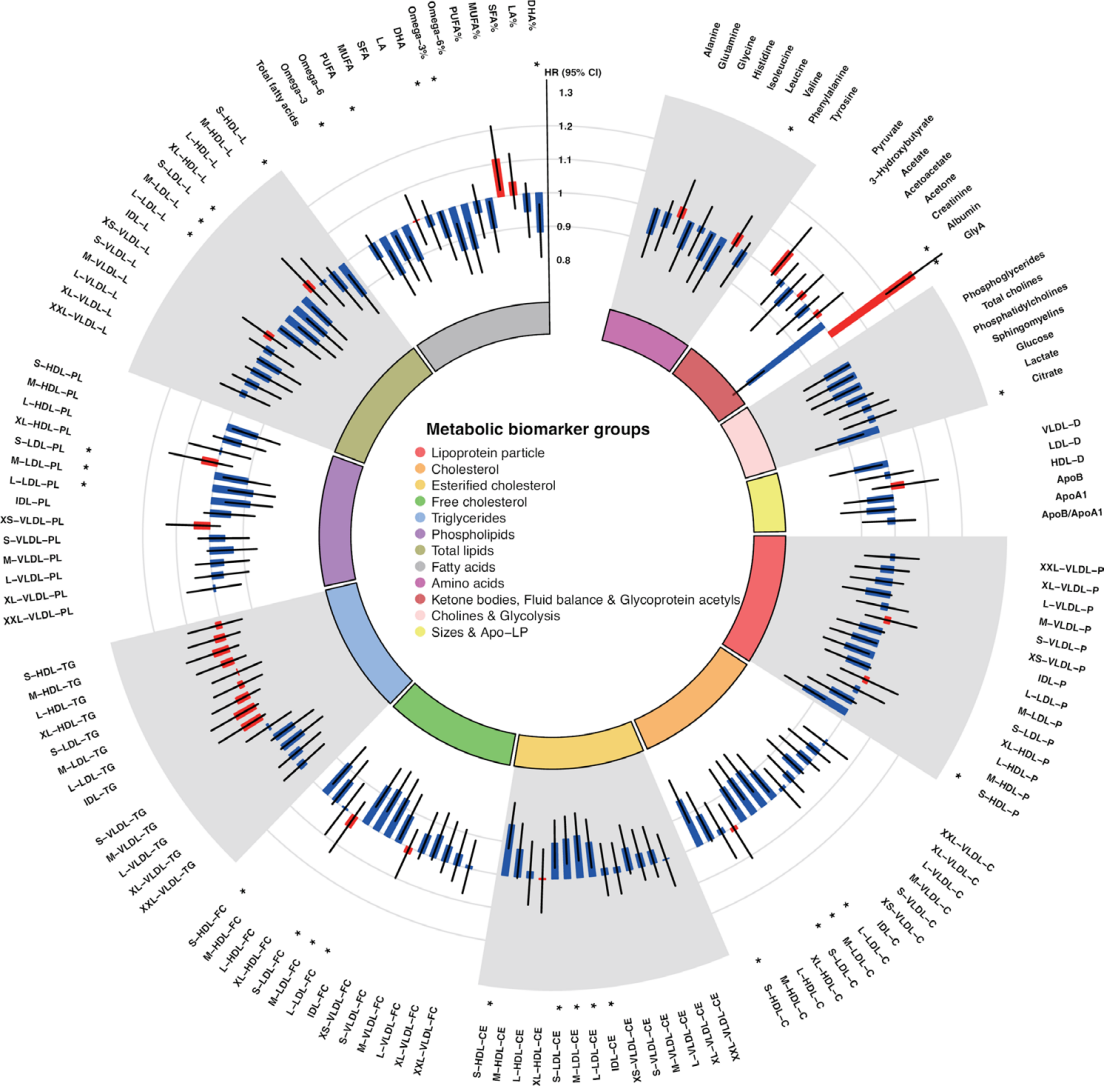
Association between baseline metabolic biomarker concentrations and the risk of incident rheumatoid arthritis in the UK Biobank study (N=93 800; 620 incident events). HRs are present per 1-SD increment in biomarker levels. Statistical models are adjusted for age, sex, UK Biobank assessment centre, fasting time, education level, smoking status, body mass index and physical activity duration. Red points denote harmful effects and green points have protective effects. *False discovery rate-controlled p<0.05. ApoA1, apolipoprotein A1; ApoB, apolipoprotein B; C, cholesterol; CE, cholesteryl ester; DHA, docosahexaenoic acid; FC, free cholesterol; HDL, high-density lipoprotein; IDL, intermediate-density lipoprotein; L, total lipids; L, large; LA, linoleic acid; LDL, low-density lipoprotein; M, medium; MUFA, monounsaturated fatty acid; P, particle concentration; PG, phosphoglycerides; PL, phospholipids; PUFA, polyunsaturated fatty acid; S, small; SFA, saturated fatty acid; TG, triglycerides; VLDL, very low-density lipoprotein; XL, very large; XS, very small; XXL, extremely large.

The mean concentrations, SDs and HRs of the 143 metabolic biomarkers associated with RA are listed in online supplemental table 1. After adjusting for age, sex, UKB assessment centre, fasting time, education level, smoking status, BMI and the physical activity duration, a total of 30 of the 143 metabolic biomarkers showed significant associations with future RA events in the Cox models at FDR-controlled p<0.05 (figure 1). Among those metabolites, glycoprotein acetyls (GlycA) (HR=1.31, 95% CI 1.21 to 1.41) were associated with an increased risk of RA. Conversely, albumin (HR=0.72, 95% CI 0.66 to 0.78) showed a most significant protective effect on the incidence of RA. Metabolites associated with decreased incidence of RA also included concentration of small HDL particles and the cholesteryl and lipids within those particles, cholesteryl ester in intermediate-density lipoprotein, as well as the cholesteryl, phospholipids and lipids in the LDL across the different sizes. In relation to fatty acids, the levels of polyunsaturated fatty acids (PUFAs), omega-3, docosahexaenoic acid (DHA), and the percentages of omega-3 and DHA to total fatty acids were inversely associated with the RA risk. Besides, valine (HR=0.89, 95% CI 0.82 to 0.97) and citrate (HR=0.88, 95% CI 0.80 to 0.95) were also identified to be negatively associated with RA risk.

**Figure 2:**
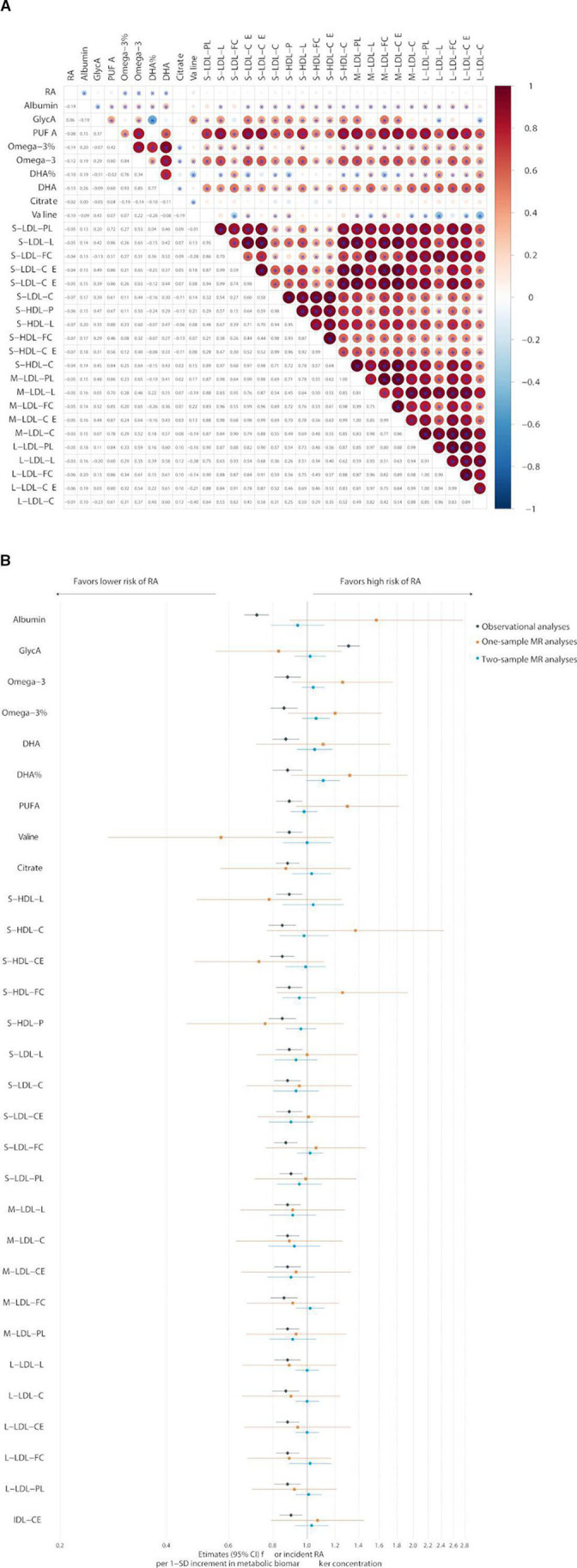
Genetic analyses between 30 candidate metabolic biomarkers and RA. (A) Cross-trait genetic correlation matrix between 30 candidate metabolic biomarkers and RA. (B) Comparison of the causal estimates of associations between 30 candidate metabolic biomarkers and RA from the observational, one-sample and two-sample Mendelian randomisation analyses. C, cholesterol; CE, cholesteryl ester; DHA, docosahexaenoic acid; FC, free cholesterol; HDL, high-density lipoprotein; IDL, intermediate-density lipoprotein; L, total lipids; L, large; LDL, low-density lipoprotein; M, medium; P, particle concentration; PL, phospholipids; PUFA, polyunsaturated fatty acid; RA, rheumatoid arthritis; S, small.

Then, genetic correlation analysis was conducted using LDSC regression to identify the genetic-wide genetic overlap between candidate metabolites and RA. Then, we found evidence for a shared genetic basis of albumin (r_g_=−0.19, p*=*1.01×10^−5^), omega-3 (r_g_=−0.12, p*=*0.004), DHA (r_g_=−0.13, p*=*0.003), and the percentages of omega-3 (r_g_=−0.14, p*=*0.004) and DHA (r_g_=−0.10, p*=*0.025) to total fatty acids with RA (figure 2A and online supplemental table 10). Motivated by the genetic correlations, we used one-sample and two-sample MR analyses to explore the causal relationship between candidate metabolites and RA. However, we did not find any strong evidence supporting the notion of a causal relationship between the candidate metabolites and RA (figure 2B and online supplemental tables 11 and 12).

**Figure 3:**
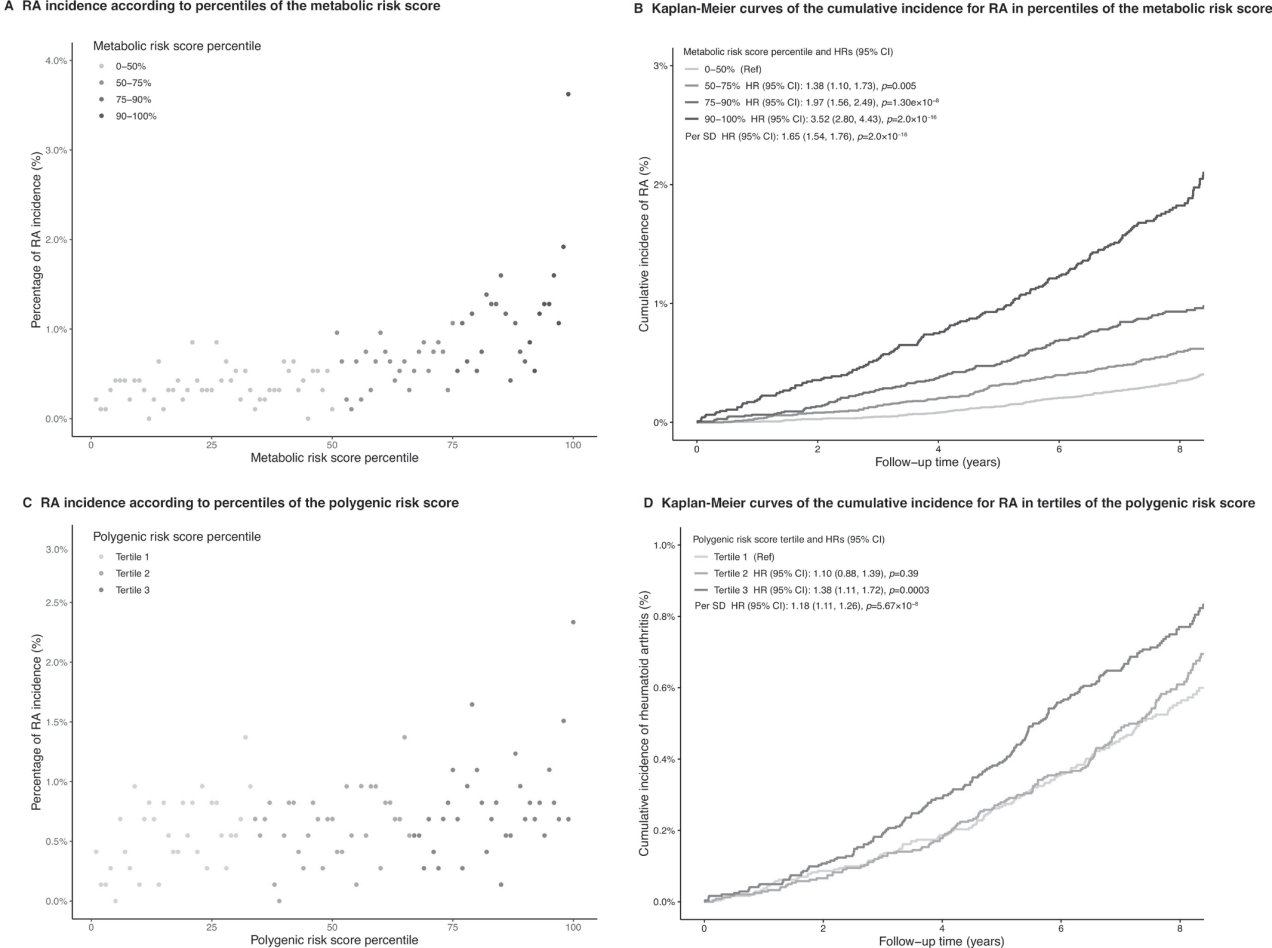
Risk gradient for incident RA according to the metabolic risk score or polygenic risk scores. (A) Proportion of individuals who were incident RA during a median follow-up time of 8 years after the blood sampling according to percentiles of the metabolic risk score. (B) Kaplan-Meier curves of the cumulative incidence for RA in percentiles of the metabolic risk score. (C) Proportion of individuals who were incident RA during a median follow-up time of 8 years after the blood sampling according to tertiles of the polygenic risk scores. (D) Kaplan-Meier curves of the cumulative incidence for RA in percentiles of the polygenic risk scores. Each point represents approximately 500 individuals. The follow-up time was truncated at 9 years since only a small fraction of individuals was followed longer. RA, rheumatoid arthritis.

Considering the limited impact of individual metabolites on discerning individuals at elevated risk of RA, we proceeded to construct a composite MRS and investigated its association with RA risk. As shown in figure 3, the risk of developing RA increased monotonically with the percentile of the MRS, and individuals in the top decile of MRS having an increased risk of RA (HR 3.53, 95% CI: 2.80 to 4.43) compared with those in below median of MRS (figure 3A,B). Broadly similar results were obtained using all 143 metabolic measures quantified in the Nightingale Health NMR platform to derive the MRS (online supplemental table 13).

**Figure 4:**
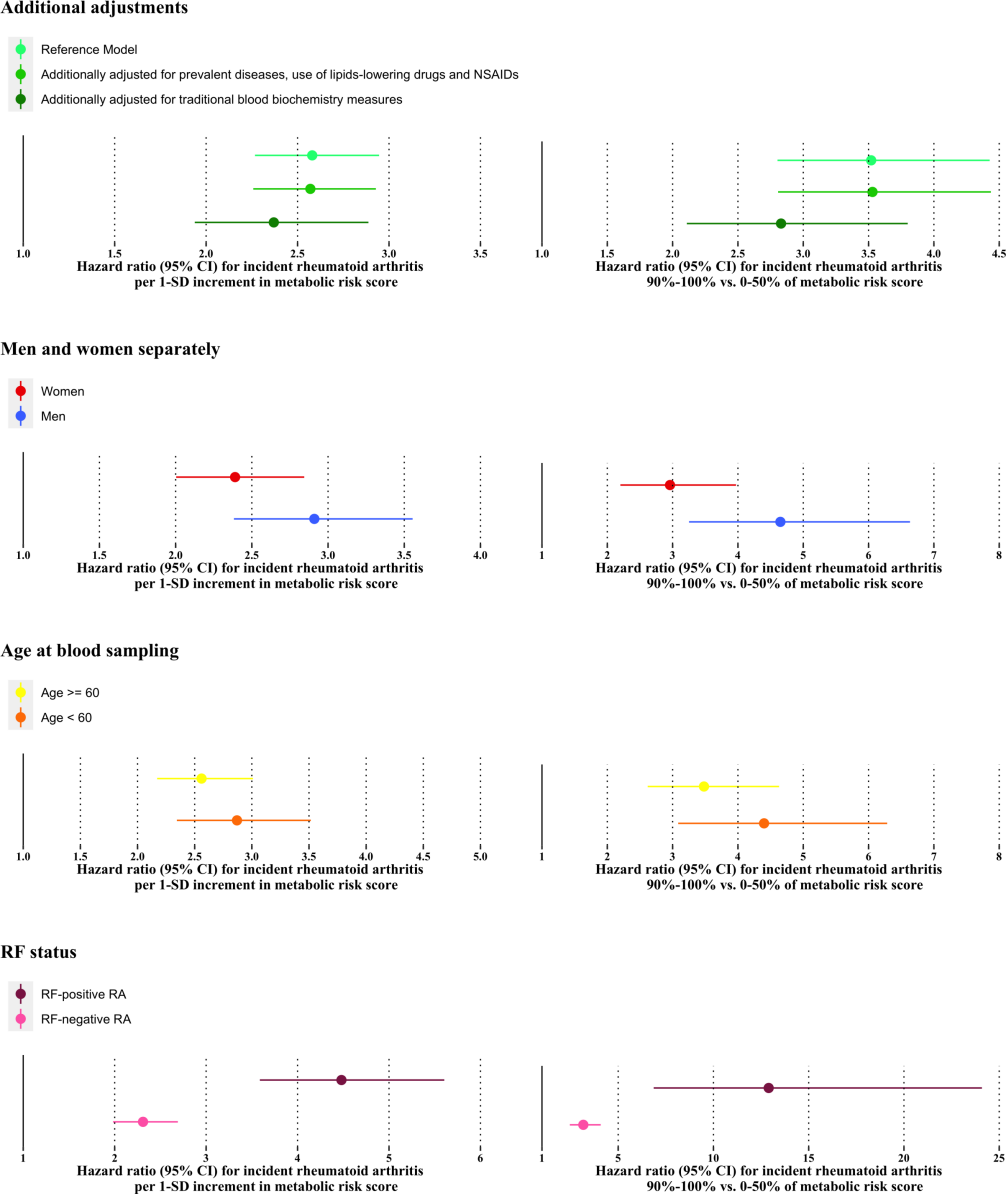
Association of the metabolic risk score with incident rheumatoid arthritis (RA) with additional adjustments and subgroups. The reference model adjusted for age, sex, UK Biobank assessment centre, fasting time, education level, smoking status, body mass index and duration of physical activity. The subgroup of women adjusted the use of hormone replacement therapy and menopause status. NSAIDs, non-steroidal anti-inflammatory drugs; RF, rheumatoid factor.

We further checked the robustness of the association between the MRS and RA risk through additional adjustments and conducted stratified analyses for age, sex and rheumatoid factor status (figure 4 and online supplemental table 14). The association with RA risk was similar after additionally adjusted for prevalent diseases (CVD, cancer, diabetes), lipid-lowing drugs and NSAIDs taken, but was attenuated by nearly 20% in magnitude after further adjusted for traditionally clinical blood measures (albumin, HDL-cholesterol, LDL-cholesterol, triglycerides, C reactive protein). Subgroup analyses revealed a more substantial effect on RA risk in individuals who were men, aged ≤60 years old and had positive rheumatoid factor status (figure 4, online supplemental table 14 and online supplemental figure 4). The MRS also showed stronger relation to long-term (>5 years) RA risk, with an HR per SD increment of 2.22 (95% CI 1.83 to 2.69) and 1.44 (95% CI 1.16 to 1.80) for individuals when compared with short-term RA risk (online supplemental table 15). In addition, the association was similar when excluding incident RA cases during the first 2 years of follow-up (online supplemental table 16).

Moreover, the joint distribution of MRS and PRS of RA was illustrated in this study (online supplemental figure 5) to improve risk stratification or probe potential gene–environment interactions. The PRSs of RA were significantly associated with elevated RA risk, with HR per SD increment of 1.18 (95% CI 1.11 to 1.26). Similarly, participants in the top tertile PRS group of RA had a 38% higher risk of RA than those in the bottom tertile group (figure 3C,D).

**Figure 5:**
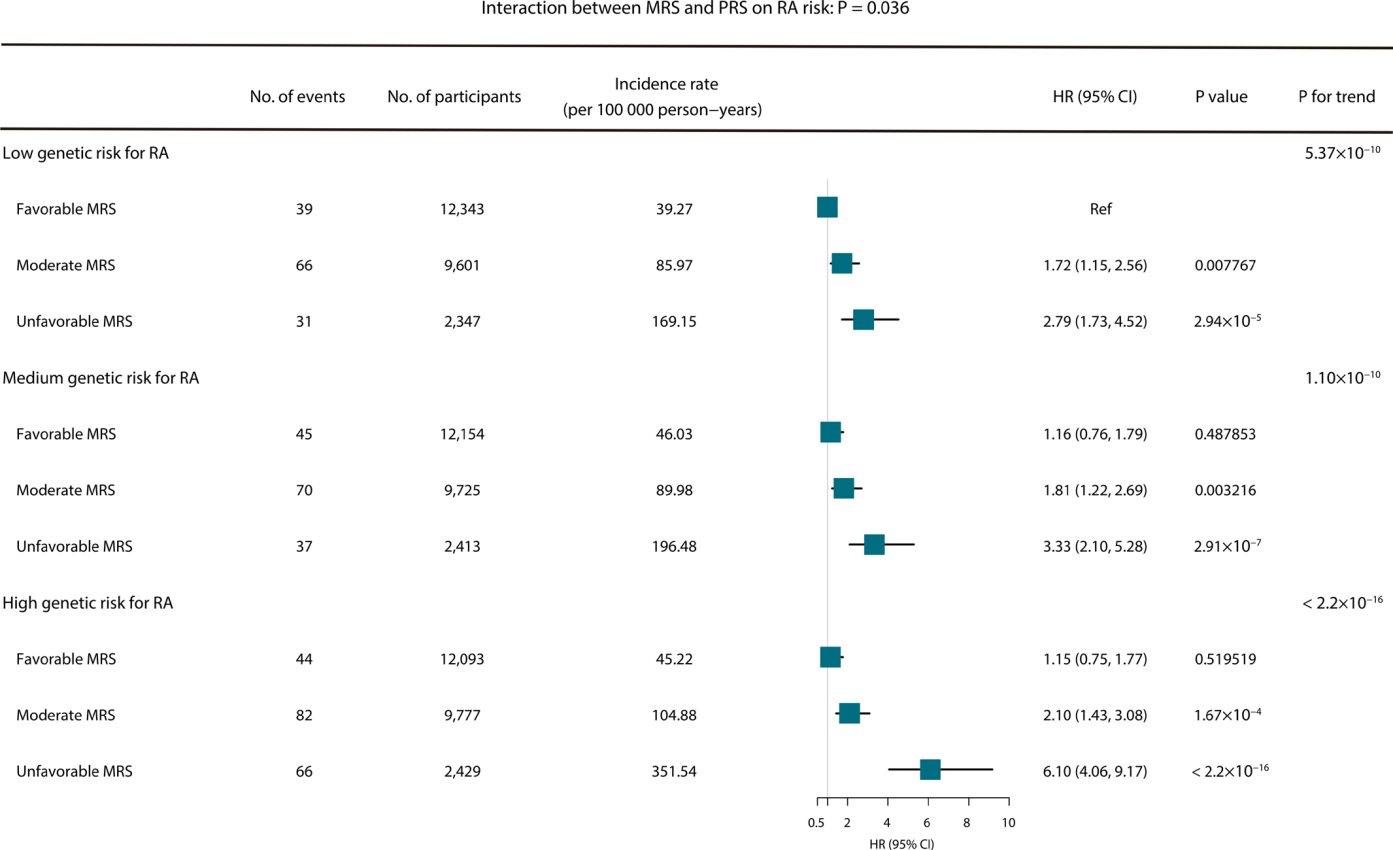
Joint associations of polygenic risk score (PRS) and metabolic risk score (MRS) with incident rheumatoid arthritis (RA). MRS (favourable: 0–50%, moderate: 50–90% and unfavourable: 90–100%) and genetic risk (low: tertile first of PRS, medium: tertile second of PRS, high: tertile third of PRS). Adjusted for age, sex, first 10 genetic principal components and genotyping batch, UK Biobank assessment centre, fasting time, education level, smoking status, body mass index and physical activity duration.

The joint association of RA metabolic and genetic predisposition with the risk of incident RA was further examined (figure 5). Significant gradient associations between MRS and RA risk were observed across genetic risk strata; specifically in the high genetic risk strata, compared with participants with favourable MRS, the risk of incident RA for participants with unfavourable MRS almost elevated by fivefold (incidence rate: 351.54 per 100 000 person-years, HR 6.10, 95% CI: 4.06 to 9.17). Also, a multiplicative interaction was found between MRS and PRS (p=0.036).

## Discussion

Using data from 93 800 participants with NMR metabolic biomarker profiling from the UKB, we performed the hitherto most extensive prospective cohort study to uncover an advanced understanding of the association between metabolomics and RA risk.

We discovered that 30 serum metabolites spanning multiple molecular pathways, including fatty acids, body fluid balance, glycolysis and detailed lipoprotein particle composition, were significantly associated with future RA events. GlycA was elevated in participants who later developed RA within the cohort study. In line with this, previous studies have demonstrated that GlycA was a predictive biomarker for long-term risk of mortality, type 2 diabetes and CVD events.^28–30^ Also, it has been identified as an innovative inflammatory biomarker that holds the potential for evaluating disease activity and its association with coronary artery atherosclerosis in patients with RA.^31^ The elevation of GlycA in apparently healthy individuals was related to elevated levels of myriad inflammatory cytokines and increased neutrophil activity, suggesting that those individuals may be in a chronic inflammatory response state.^32^ In contrast, in this study, we observed negative correlations between small HDL, LDL and its lipid constituents and incident RA. Dyslipidaemia is a common phenomenon of RA and occurs in 55–65% of patients with RA.^33 34^ Moreover, dyslipidaemia was found to exist before RA was diagnosed. A previous retrospective study of blood donors has shown that patients who developed RA after 10 years have average higher levels of total cholesterol and lower levels of HDL-cholesterol compared with controls.^35^ Another longitudinal population-based study also found that patients with RA during the 5 years prior to diagnosis^36^ had a 17% reduction in LDL levels compared with subjects without RA. The declining trend in HDL and LDL levels in this study is concordant with our present analysis.

In accordance with previous findings,^37 38^ inversely, associations between PUFA, omega-3 and DHA were observed with RA incidence in our study. Similarly, the Swedish Mammography Cohort, which includes 32 232 female participants, has identified a potential protective effect of omega-3 PUFAs against RA risk.^39^ PUFA, omega-3 and DHA are well-known for their inflammation-suppressing functions,^13^ and current animal models and inflammatory cell function experiments provide some insight into the mechanisms that may be involved. Specifically, increased intake of eicosapentaenoic acid (EPA) and DHA increases the content of EPA and DHA in inflammatory cell membranes, partially replacing arachidonic acid (AA) and thereby reducing AA availability for phosphoglyceride synthesis.^40^

The inverse assocaitiona of valine, citrate, and albumin and RA risk were also detected in our study. The metabolite pathway analysis was previously used to identify biomarker changes and found that the valine level was lower in patients with RA^12^ and to be increased after etanercept treatment.^41^ In addition, hypoalbuminaemia is highly detected in patients with RA and is strongly related to the disease activity of RA.^42^ Inflammation is the leading cause of hypoalbuminaemia, as interleukin 1 can hinder the expression of messenger RNA and the synthesis of albumin.^43^ A similar inverse association was also observed between citrate and C reactive protein level in newly presenting patients with RA.^44^ The inverse correlation between citrate and RA may be related to immunometabolic reprogramming.^45^ Activation of innate immune cells such as macrophages and dendritic cells leads to the upregulation of glycolysis and downregulation of the citric cycle.^46^

Considering the limited risk estimation of a single metabolite for RA and from a translational medicine perspective, we further used 17 clinically validated metabolic markers to construct an MRS to probe the relatively comprehensive metabolomic influence on RA risk. To our knowledge, the previous study has largely ignored the interactions between genetics and metabolomics. We thus constructed a PRS to represent the overall genetic risk of RA to explore their interaction effects. We observed a significant interaction effect along with the high genetic risk of RA and high MRS synergistically enhanced the risk of developing RA. The observed findings can identify individuals most susceptible to RA and target them with precise interventions.

Our study has several strengths. First, to our current knowledge, this is the first metabolomics study that uses large samples with a prospective cohort study design on RA. Second, we conducted an MR analysis to further elucidate the causal effect between metabolic biomarkers and RA. Third, the contribution of genetic factors to the association between metabolic profiles and RA was assessed, providing insights into the new prevention strategies.

This study has certain limitations which should be considered. The cohort of participants was predominantly composed of European ancestry and those with higher socioeconomic status, which may have introduced selection bias and limited the results’ generalisability to populations of diverse ethnicities and varying economic backgrounds. Besides, lifestyle patterns may affect the metabolomic perturbation^47^; the underlying biological mechanisms of complex interactions on development of RA still need to be elucidated. Moreover, we could not replicate the association of metabolic biomarkers with incident RA found in previous studies,^25 26^ especially the lack of biomarkers related to sex hormones, which influence immune cell development and have immunomodulatory effects.^48 49^ Furthermore, with regard to potential genetic and metabolic pathway differences that might exist among different ACPA (anti-cyclic citrullinated peptide antibodies)-RA serological types,^50 51^ we could not investigate this heterogeneity due to the unavailability of ACPA data in the UKB. Last, it is essential to acknowledge that the relatively modest sample size of the current metabolite GWAS may hinder the evaluation of moderate or low metabolite–RA causal associations. This limitation renders the MR analyses potentially underpowered in our study.

## Conclusions

Although our findings indicated a scarcity of evidence supporting a causal link between individual metabolic biomarkers and RA, the composite MRS, which encompasses multiple metabolic biomarkers, effectively identifies an increased susceptibility to RA. Moreover, the integration of metabolic profiles and genetic predisposition enhances risk stratification, thus facilitating personalised prevention strategies for RA.

## Data Availability

Data are available upon reasonable request. The UK Biobank data are available on application to the UK Biobank to any researcher worldwide (www.ukbiobank.ac.uk). Summary statistics of used and/or analysed during the study are available from the corresponding author (D-QY) upon reasonable request.
