# Supplementary material for: Metabolomic profiles, polygenic risk scores and risk of rheumatoid arthritis: a population-based cohort study in the UK Biobank
Source: RMD Open. 2023 Nov 30;9(4):e003560. doi: 10.1136/rmdopen-2023-003560 (PMC10689387; doi:10.1136/rmdopen-2023-003560)
Supplement: Supplementary data [file rmdopen-2023-003560supp001.pdf]

Content

Supplementary Methods

Text S1 Genotyping and imputation

Text S2 Mendelian randomization analyses

Text S3 Construction of polygenic risk score for RA

Supplementary Tables

Table S1 The distribution of 143 metabolic biomarkers and the hazard ratios of incident RA.

Table S2 Codes used in the UK Biobank study to identify RA cases.

Table S3 Tests for weak instrument bias and heterogeneity of instrumental variables in the two-sample MR analyses.

Table S4 Tests for weak instrument and statistical power in the one-sample MR analyses.

Table S5. Non-MHC region SNPs used to construct PRS

Table S6. Effect estimates for the five amino acids of haplotypes of *HLA-DRB1*, *HLA*-B and *HLA-DPB1* associated with RA Risk.

Table S7 The weighted coefficients of metabolic risk score based the 37 clinically validated biomarkers.

Table S8 The weighted coefficients of metabolic risk score based the 143 NMR-quantified metabolites

Table S9 The basic characteristics of participants with and without metabolic biomarker profiling.

Table S10 Cross-trait genetic correlation between candidate metabolic biomarkers and RA.

Table S11 The one and two-sample MR analyses on casual relationship of candidate metabolic biomarkers with RA.

Table S12 Sensitivity analyses for two-sample MR analyses.

Table S13 Associations of metabolic risk score with the risk of RA based on the 37 clinically-validated metabolite sets and all 143 measured metabolite sets.

Table S14 Associations of the metabolic risk score with the risk of RA with additional adjustments and subgroups.

Table S15 Associations of the metabolic risk score with the short-term (2-5 years) and long-term (>5 years) risk of RA.

Table S16 Associations of the metabolic risk score with the risk of RA after excluding incident RA in the first 2 following years.

Supplementary Figures

Figure S1 A flow diagram of eligible study participants and overall study design.

Figure S2 The performance of metabolic risk score in the test dataset and the examination of possible time-varying effects.

Figure S3 Spearman correlation coefficients between metabolic risk score and traditional blood biochemistry measures.

Figure S4 Cumulative incidence of RA among subgroups according to gender, age and RF-status.

Figure S5 Joint distribution of metabolic risk score and polygenetic risk score.

Supplementary Methods

Text S1 Genotyping and Imputation

The genotypes of the UK Biobank participants were assayed using either of two genotyping arrays, the Affymetrix UK BiLEVE Axiom (approximately 50,000 participants) or Affymetrix UK Biobank Axiom genotyping array (approximately 450,000 participants), there are 805,426 markers in the released genotype data. These arrays were augmented by imputation of approximately 90 million genetic variants from the Haplotype Reference Consortium and the UK 10K projects. Details of the array design, sample processing, and stringent quality control have been previously described.[1] More details on genotyping and imputation sees https://biobank.ndph.ox.ac.uk/ukb/label.cgi?id=263

Text S2 Mendelian randomization analyses

In our current study, we performed two-sampe and one-sample Mendelian randomization (MR) analyses to investigate the causal relationship between candidate metabolites and RA risk. The candidate metabolites included 30 significant metabolities ascertained by our observational analyses.

For the two-sample MR analyses: the GWAS of metabolites was derived from the previous study[1] which focused on the role of circulating polyunsaturated fatty acids on cardiovascular diseases risk in the UK Biobank. The study encompassing 115,078 European particiapants, with 12,321,875 genotyping SNPs, and the download website is https://gwas.mrcieu.ac.uk/datasets/?gwas_id__icontains=met-d; for the GWAS summary statistics of RA, we combined two independent sources including the RA GWAS meta-analysis[2] (European ancestry,14,361 RA cases, 43,923 controls, 13,108,512 genotyping SNPs) and FinnGen biobank analysis[3] round 5 (European ancestry, 6,236 RA cases, 147,221 controls, 16,380,169 genotyping SNPs). And the downloaded webiste is https://gwas.mrcieu.ac.uk/datasets/ieu-a-832/ and https://gwas.mrcieu.ac.uk/datasets/finn-b-M13_RHEUMA/. It is worth noting that there was no population overlap between GWASs of exposures and outcomes.

A series of steps were conducted to select eligible SNPs as instrumental variables (IVs). Firstly, only SNPs that were associated with each metabolite with genome-wide significance ($P < 5 \times 10^{-8}$) were selected. Considering that instrumental SNPs in strong LD may cause biased results, we applied the clumping process with an r^2 cutoff of 0.001with the European samples from the 1000 genomes

project to estimate LD between SNPs. Only the SNP with the lower *P* value would be retained among those SNPs with LD r^2 above the specified threshold. SNPs absent from the LD reference panel were also removed. Also, SNPs with minor allele frequency (MAF) < 0.3 were removed. Then, we extracted data for the above-selected SNPs from the outcome trait (RA) GWAS summary statistics. By default, if a particular requested SNP was not present in the outcome GWAS, then an SNP (proxy) that was in LD r^2 >0.8 with the requested SNP (target) would be searched for instead. The effect of the proxy SNP on the outcome was returned, along with the proxy SNP, the effect allele of the proxy SNP, and the corresponding allele (in phase) for the target SNP. Besides, the effect of ambiguous SNPs with non-concordant alleles (e.g., A/G vs. A/C) and palindromic SNPs with an ambiguous strand (i.e., A/T or G/C) was corrected or directly excluded from the above-selected instrument SNPs in harmonizing process, which aims to ensure the effects of the same SNP on exposure and outcome correspond to the same allele.All the above-selected SNPs were used as IVs in the subsequent two-sample MR analyses.

Then, we used the inverse variance weighted (IVW) method[4] as the primary two-sample MR analysis, which uses a meta-analysis approach to combine Wald estimates for each SNP (i.e., the β coefficient of the SNP for RA divides by the β coefficient of the SNP for metabolites) to get the overall estimates of the effect of metabolites on RA[4]. If there is no violation of the IV2 assumption (no horizontal pleiotropy), or the horizontal pleiotropy is balanced, an unbiased causal estimate can be obtained by IVW linear regression[5]. Moreover, as sensitivity analyses for robust causal inference, we additionally performed MR analyses using MR-Egger method[6] and weighted median (WM)[7] method. The MR-Egger regression, based on the assumption of the Instrument Strength Independent of Direct Effect (InSIDE), performs a weighted linear regression of the outcome coefficients on the exposure coefficients[6]. Under the InSIDE assumption, it gives a valid test of the null causal hypothesis and a consistent causal effect estimate even when all the genetic variants are invalid IVs[6]. However, MR-Egger estimates may be inaccurate and can be strongly influenced by outlying genetic variants. The WM estimate, which does not require the InSIDE assumption, has been confirmed to have distinct superiorities over MR-Egger for its improved power of causal effect detection and lower type I error[7]. We put the IVW method results as the main findings in the text, and the causal effects estimated were obtained by integrating the independent MR results on two different outcome sources (Okada Y et al. and FinnGen biobank analysis round 5) using fixed-effect meta-analysis.

For the one-sample MR analyses: to enhance the robustness of findings derived from two-sample MR analyses, we also performed a standard one-sample MR analysis using individual-level imputed genotyping data from UK Biobank. The genetic instruments we used were genetic risk scores (GRSs), which are integrated scores that combine multiple SNPs for each metabolite by multiplying the gene dosage matrix (i.e., multiple SNPs and their number of effective alleles, 0, 1, 2) with the effect sizes of the genome-wide association studies (GWAS). The selection of SNPs for GRSs remained the same as the instrumental variables (IVs) used in the two-sample MR analyses. F-statistics were calculated using the equation $F = R^2(n-2)/(1-R^2)$ for the one-sample MR analysis. In this formula, R^2 is the proportion of the variability in each metabolite explained by its the corresponding GRS and n is the sample size. Then, we conducted a two-stage regression[8]. In the first stage, we obtained predicted values of metabolites by regressing them on genetic instruments using linear regression. Then, we regressed the RA on the predicted values from the first stage by using a Cox regression. The covariates including age, sex, first ten genetic principal components genotyping batches and UK Biobank assessment centers were adjusted in both stages.

Text S3 Construction of polygenic risk score for RA

The data we used for constructing polygenic risk score (PRS) are UK Biobank genotype data, which can be downloaded from the European Genome-phenome Archive. The genotype calling, quality control (QC), phasing, and imputation were performed centrally and have been described in detail previously.[9] In brief, about 50,000 and 450,000 participants were separately run on the UK BiLEVE Axiom array and UK Biobank Axiom array, which are two closely related purpose-designed arrays. The dataset combines results from both arrays and there are 805,426 markers in the released genotype data. In addition, the dataset was phased and 96 million genotypes were imputed using computationally efficient methods combined with the Haplotype Reference Consortium and UK10K haplotype resources.

Based on the above genotyping data, we carried out downstream quality control measures to obtain the population that meets the purpose of our study. Firstly, we have maintained the genetic homogeneity of the study population by limiting it to European ancestry (self-reported British White and further confirmed as Caucasians in ancestry background principal component analysis). Then,

related individuals also were removed based on genetic relatedness (third-degree or greater-related). In addition, samples identified as outliers in heterozygosity and missing rates, and samples of genetic gender and self-reported gender inconsistency and sex chromosome aneuploidy were also excluded.

We comprehensively considered the genetic loci in the non-MHC genetic loci and amino acid positions in the MHC region that characterize the genetic susceptibility of RA based on previously published studies[2,10-14]. **For the calculation of $PRS_{non\text{-}MHC}$,** the summary statistics of RA GWAS were based on the largest GWAS preformed by Okada and colleagues[2], which identified 58 previously reported non-MHC RA-risk loci and 42 novel RA risk loci in individuals of European ancestry at a genome-wide level of significance.We followed several steps to choose SNPs. We first removing the SNPs located in the MHC region (chromosome 6, 25–35 Mbp). Then, we applied the clumping and thresholding ($P<5\times10^{-8}$ and $r^2<0.1$) method to further select the SNPs which would be included in the calculation of the $PRS_{non\text{-}MHC}$. Additionally, if the selected variants were not present in UK biobank genotyping data, then we will seek proxy variants ($r^2>0.8$). Finally, we obtained 58 SNPs be included in $PRS_{non\text{-}MHC}$. The $PRS_{non\text{-}MHC}$ in this study was cauclated based on the additive genetic model, and the final PRS is standardized. The calculation formula is: $PRS_j=[\sum_i (S_i\times G_{ij})\text{-mean}(PRS)]/\text{SD}(PRS)$, S is the summary statistic for the effective allele and G is the number of the effective allele observed, i is ith SNPs and j is jth individuals.

For the calculation of PRS_{HLA}, we included three amino acid positions (11, 71 and 74) in *HLA-DRB1* and single–amino-acid polymorphisms in *HLA-B* (at position 9) and *HLA-DPB1* (at position 9)[10-12.] These postions are all located in peptide-binding grooves, and were demonstrated can almost completely explain the MHC association to RA risk[10-12]. We downloaded imputed classical allele genotypes for *HLA-A, HLA-B, HLA-C, HLA-DPA1, HLA-DPB1, HLA-DQA1, HLA-DQB1* and *HLA-DRB1*, and their corresponding amino acid sequences and SNPs within the MHC from the UK Biobank after approval (https://biobank.ndph.ox.ac.uk/ukb/label.cgi?id=100035). In brief, imputation of four-digit HLA alleles from SNPs was carried out using HLA*IMP:02 (the further information and QC can be seen in https://biobank.ndph.ox.ac.uk/ukb/ukb/docs/HLA_imputation.pdf). We then constructed PRS_{HLA} in the case of the imputed HLA amino acids, and each haplotype was weighted by published[12] odds ratio.The calculation formula is: $PRS_{HLAj}=[\sum_i (OR_i\times G_{ij})\text{-mean}(PRS_{HLA})]/\text{SD}(PRS_{HLA})$, G is the number of the HLA alleles observed, i is ith SNPs and j is jth individuals.

In the end, we summed the $PRS_{non\text{-}MHC}$. and PRS_{HLA} into a full PRS to represent comprehensive genetic disposition of RA. The $PRS_{non\text{-}MHC}$ calculation was performed in PRSice-2[15] with RA GWAS statistics and the imputed genotype data from UK Biobank.

References

1. Borges MC, Haycock PC, Zheng J, et al. Role of circulating polyunsaturated fatty acids on cardiovascular diseases risk: analysis using Mendelian randomization and fatty acid genetic association data from over 114,000 UK Biobank participants. BMC Med. 2022;20(1):210. Published 2022 Jun 13. doi:10.1186/s12916-022-02399-w
2. Okada Y, Wu D, Trynka G, et al. Genetics of rheumatoid arthritis contributes to biology and drug discovery. Nature. 2014;506(7488):376-381. doi:10.1038/nature12873
3. Kurki M.I., et al. FinnGen: Unique genetic insights from combining isolated population and national health register data, medRxiv 2022.03.03.22271360
4. Burgess S, Dudbridge F, Thompson SG. Combining information on multiple instrumental variables in Mendelian randomization: comparison of allele score and summarized data methods. Stat Med. 2016;35(11):1880-1906. doi:10.1002/sim.6835
5. Hemani G, Zheng J, Elsworth B, et al. The MR-Base platform supports systematic causal inference across the human phenome. Elife. 2018;7:e34408. Published 2018 May 30. doi:10.7554/eLife.34408
6. Bowden J, Davey Smith G, Burgess S. Mendelian randomization with invalid instruments: effect estimation and bias detection through Egger regression. Int J Epidemiol. 2015;44(2):512-525. doi:10.1093/ije/dyv080
7. Bowden J, Davey Smith G, Haycock PC, Burgess S. Consistent Estimation in Mendelian Randomization with Some Invalid Instruments Using a Weighted Median Estimator. Genet Epidemiol. 2016;40(4):304-314. doi:10.1002/gepi.21965
8. Richmond RC, Anderson EL, Dashti HS, et al. Investigating causal relations between sleep traits and risk of breast cancer in women: mendelian randomisation study. BMJ. 2019;365:l2327. Published 2019 Jun 26. doi:10.1136/bmj.l2327
9. Bycroft C, Freeman C, Petkova D, Band G, Elliott LT, Sharp K, Motyer A, Vukcevic D, Delaneau O, O'Connell J, Cortes A, Welsh S, Young A, Effingham M, McVean G, Leslie S, Allen N, Donnelly P, Marchini J. The UK Biobank resource with deep phenotyping and genomic data. Nature. 2018 Oct;562(7726):203-209. doi: 10.1038/s41586-018-0579-z
10. Lenz TL, Deutsch AJ, Han B, et al. Widespread non-additive and interaction effects within HLA loci modulate the risk of autoimmune diseases. Nat Genet. 2015;47(9):1085-1090. doi:10.1038/ng.3379
11. Kim K, Bang SY, Lee HS, Bae SC. Update on the genetic architecture of rheumatoid arthritis. Nat Rev Rheumatol. 2017;13(1):13-24. doi:10.1038/nrrheum.2016.176
12. Raychaudhuri S, Sandor C, Stahl EA, et al. Five amino acids in three HLA proteins explain most of the association between MHC and seropositive rheumatoid arthritis. Nat Genet. 2012;44(3):291-296. Published 2012 Jan 29. doi:10.1038/ng.1076
13. Yarwood A, Han B, Raychaudhuri S, et al. A weighted genetic risk score using all known susceptibility variants to estimate rheumatoid arthritis risk. Ann Rheum Dis. 2015;74(1):170-176. doi:10.1136/annrheumdis-2013-204133

14. Bouzit L, Malspeis S, Sparks JA, et al. Assessing improved risk prediction of rheumatoid arthritis by environmental, genetic, and metabolomic factors. Semin Arthritis Rheum. 2021;51(5):1016-1022. doi:10.1016/j.semarthrit.2021.07.006

15. Choi SW, O'Reilly PF. PRSice-2: Polygenic Risk Score software for biobank-scale data. Gigascience. 2019;8(7):giz082. doi:10.1093/gigascience/giz082

Table S1 The distribution of 143 metabolic biomarkers and the hazard ratios of incident RA.

Metabolites	Class	Mean	sd	HR	HR_lower	HR_upper	FDR_P_value
VLDL-D	Sizes & Apo-LP	38.69430171	1.206888652	0.900236291	0.824276203	0.983196382	0.080660592
LDL-D	Sizes & Apo-LP	23.91516728	0.087549284	0.986853022	0.909370994	1.070936826	0.850326057
HDL-D	Sizes & Apo-LP	9.634919759	0.20046192	1.036795557	0.942663399	1.140327531	0.628120905
Phosphoglycerides	Cholines & Glycolysis	2.248546696	0.393202311	0.917650939	0.843543278	0.998269168	0.141338336
Total cholines	Cholines & Glycolysis	2.525731925	0.405613163	0.91089566	0.836764159	0.991594698	0.114289707
Phosphatidylcholines	Cholines & Glycolysis	2.072000393	0.370181187	0.912381821	0.837820052	0.993579212	0.122154548
Sphingomyelins	Cholines & Glycolysis	0.440697173	0.070833681	0.941416421	0.865205332	1.024340517	0.324313626
ApoB	Sizes & Apo-LP	0.845060478	0.198077236	0.923997994	0.854161625	0.999544193	0.148116163
ApoA1	Sizes & Apo-LP	1.429682908	0.23466845	0.919440975	0.840787632	1.005452119	0.17794794
ApoB/ApoA1	Sizes & Apo-LP	0.604563965	0.165148116	0.979496173	0.904105464	1.061173492	0.781608232
Total fatty acids	Fatty acids	11.89872924	2.375937392	0.950945074	0.877457047	1.030587806	0.420015932
**Omega-3**	**Fatty acids**	**0.525619877**	**0.217880261**	**0.876240887**	**0.803515187**	**0.955548949**	**0.019086589**
Omega-6	Fatty acids	4.461600998	0.67867129	0.907435838	0.836149428	0.984799812	0.080660592
**PUFA**	**Fatty acids**	**4.987220754**	**0.798218671**	**0.888287478**	**0.817613297**	**0.965070708**	**0.029604273**
MUFA	Fatty acids	2.847182759	0.811627482	1.000712805	0.924035168	1.083753253	0.993368192
SFA	Fatty acids	4.064327036	0.945912599	0.968595823	0.89446281	1.048872975	0.605926888
LA	Fatty acids	3.426482198	0.6824933	0.920246757	0.848642319	0.997892839	0.140843246
**DHA**	**Fatty acids**	**0.232627658**	**0.081957329**	**0.868812537**	**0.794348594**	**0.950256891**	**0.018247737**
**Omega-3%**	**Fatty acids**	**4.391792179**	**1.530991315**	**0.863153562**	**0.789913746**	**0.943184082**	**0.01469116**
Omega-6%	Fatty acids	37.967696	3.609285575	0.965606028	0.88817183	1.049791233	0.605869051
PUFA%	Fatty acids	42.3594873	3.734047064	0.911171471	0.837999713	0.99073238	0.110660494
MUFA%	Fatty acids	23.64026537	2.624175896	1.11206792	1.020021724	1.212420315	0.071341927
SFA%	Fatty acids	34.0002475	1.949415893	1.038112595	0.958959693	1.123798809	0.559067023
LA%	Fatty acids	28.99096319	3.442257755	0.947135629	0.870063075	1.031035479	0.412832513
**DHA%**	**Fatty acids**	**1.985455095**	**0.660376288**	**0.8839985**	**0.808524749**	**0.966517536**	**0.035856851**
Alanine	Amino acids	0.297823113	0.076302363	0.922898851	0.849962039	1.002094506	0.167158184
Glutamine	Amino acids	0.529702991	0.078693504	0.956826324	0.88215159	1.037822326	0.494623905
Glycine	Amino acids	0.16213786	0.063274672	1.029939113	0.947464642	1.119592785	0.665257411
Histidine	Amino acids	0.064300483	0.01036232	0.914818181	0.839726527	0.996624827	0.135242705
Isoleucine	Amino acids	0.050178424	0.017434707	0.983999684	0.906771107	1.067805723	0.822052514
Leucine	Amino acids	0.101294725	0.027614187	0.909967573	0.833799042	0.993094189	0.122154548
**Valine**	**Amino acids**	**0.203948822**	**0.041783256**	**0.891819613**	**0.817262422**	**0.973178506**	**0.048423779**
Phenylalanine	Amino acids	0.045216526	0.010810153	1.036787952	0.976638162	1.100642284	0.444253793
Tyrosine	Amino acids	0.061534626	0.013896659	0.949292177	0.874543878	1.0304293	0.412832513
Glucose	Cholines & Glycolysis	3.571566719	1.13094362	0.97761939	0.902553143	1.058928971	0.752298331
Lactate	Cholines & Glycolysis	3.808090773	1.081135767	0.982759048	0.90389211	1.068507331	0.822052514
Pyruvate	Ketone bodies, Fluid balance & Glycoprotein acetyls	0.078124102	0.028480104	1.081494058	0.993251721	1.177576009	0.183034434
**Citrate**	**Cholines & Glycolysis**	**0.062149366**	**0.012095838**	**0.875493703**	**0.804094377**	**0.953232912**	**0.018247737**
3-Hydroxybutyrate	Ketone bodies, Fluid balance & Glycoprotein acetyls	0.057659457	0.057554675	0.985285371	0.906748539	1.070624568	0.837364186
Acetate	Ketone bodies, Fluid balance & Glycoprotein acetyls	0.015025837	0.011140493	0.93856731	0.828831249	1.062832266	0.531159321
Acetoacetate	Ketone bodies, Fluid balance & Glycoprotein acetyls	0.012641701	0.011960814	1.019896756	0.946727789	1.098720672	0.778086366
Acetone	Ketone bodies, Fluid balance & Glycoprotein acetyls	0.014136457	0.005269864	0.957612411	0.877715622	1.044782052	0.54175293
Creatinine	Ketone bodies, Fluid balance & Glycoprotein acetyls	0.066204903	0.014034707	1.016092913	0.932543108	1.107128238	0.831670018
**Albumin**	**Ketone bodies, Fluid balance & Glycoprotein acetyls**	**38.91894294**	**3.275175657**	**0.716563589**	**0.660866512**	**0.776954753**	**9.79E-14**
**GlyA**	**Ketone bodies, Fluid balance & Glycoprotein acetyls**	**0.795494096**	**0.115262759**	**1.305023605**	**1.206451227**	**1.41164978**	**2.19E-09**
XXL-VLDL-P	Lipoprotein particle	1.77E-06	1.51E-06	0.989040122	0.911414673	1.073276952	0.87073602
XXL-VLDL-L	Total lipids	0.239385958	0.203918185	0.983362336	0.905995845	1.06733545	0.822052514
XXL-VLDL-PL	Phospholipids	0.037618796	0.031673968	0.995590633	0.917630569	1.080174029	0.962522807
XXL-VLDL-C	Cholesterol	0.058604607	0.042130887	0.99613281	0.918244763	1.080627536	0.96622348
XXL-VLDL-CE	Esterified cholesterol	0.032361333	0.02314099	0.999476614	0.921440287	1.084121799	0.993368192
XXL-VLDL-FC	Free cholesterol	0.026243279	0.019220713	0.992174388	0.914555595	1.076380727	0.913984006
XXL-VLDL-TG	Triglycerides	0.143162549	0.131863654	0.976657291	0.899567786	1.06035307	0.752266425
XL-VLDL-P	Lipoprotein particle	3.59E-06	2.24E-06	0.962964057	0.885783037	1.046870098	0.560005753
XL-VLDL-L	Total lipids	0.210069809	0.13194921	0.962275825	0.885256203	1.045996358	0.559067023
XL-VLDL-PL	Phospholipids	0.040111513	0.025590963	0.96698055	0.89005371	1.050556133	0.605869051
XL-VLDL-C	Cholesterol	0.055103691	0.027917049	0.963187005	0.887473002	1.045360484	0.559067023
XL-VLDL-CE	Esterified cholesterol	0.030613583	0.014285488	0.961291987	0.88637884	1.042536489	0.54669248
XL-VLDL-FC	Free cholesterol	0.024490109	0.014085365	0.966551293	0.890082082	1.049590169	0.605869051
XL-VLDL-TG	Triglycerides	0.11485461	0.080597161	0.961684831	0.88431185	1.04582757	0.559067023

Metabolites	Class	Mean	sd	HR	HR_lower	HR_upper	FDR_P_value
L-VLDL-L	Total lipids	0.33694577	0.171870214	0.940158814	0.864662267	1.022247216	0.303388085
L-VLDL-PL	Phospholipids	0.067975056	0.038463442	0.961924643	0.885141256	1.045368762	0.559067023
L-VLDL-C	Cholesterol	0.099717295	0.046186408	0.955588983	0.880450409	1.037139963	0.482950105
L-VLDL-CE	Esterified cholesterol	0.052194211	0.022626003	0.959918134	0.885645193	1.040419834	0.531159321
L-VLDL-FC	Free cholesterol	0.047523073	0.024234364	0.952459614	0.876280001	1.03526192	0.450670053
L-VLDL-TG	Triglycerides	0.169253472	0.090215204	0.925020524	0.849801879	1.006897008	0.183034434
M-VLDL-P	Lipoprotein particle	3.49E-05	1.20E-05	0.927925562	0.856808853	1.004945088	0.17794794
M-VLDL-L	Total lipids	0.576516239	0.200793312	0.92570079	0.854191861	1.00319611	0.171051607
M-VLDL-PL	Phospholipids	0.129081814	0.046738181	0.930949139	0.859994754	1.007757659	0.186627983
M-VLDL-C	Cholesterol	0.171099378	0.065151418	0.931394311	0.860848719	1.007721035	0.186627983
M-VLDL-CE	Esterified cholesterol	0.09181425	0.038080558	0.934159952	0.863217355	1.010932891	0.20654899
M-VLDL-FC	Free cholesterol	0.079285139	0.029026601	0.931189389	0.860440179	1.007755913	0.186627983
M-VLDL-TG	Triglycerides	0.276335017	0.112426383	0.933568101	0.859171542	1.014406735	0.230376931
S-VLDL-P	Lipoprotein particle	3.95E-05	1.26E-05	0.981264437	0.906132488	1.06262595	0.812016605
S-VLDL-L	Total lipids	0.413870186	0.127284093	0.974181094	0.899592843	1.054953706	0.694690327
S-VLDL-PL	Phospholipids	0.09733696	0.029386282	0.955265219	0.882670215	1.033830781	0.452665092
S-VLDL-C	Cholesterol	0.157345669	0.050512257	0.96878164	0.895717121	1.047806103	0.605869051
S-VLDL-CE	Esterified cholesterol	0.098701387	0.032401088	0.984039128	0.909906507	1.064211539	0.822052514
S-VLDL-FC	Free cholesterol	0.05864428	0.018510238	0.943210259	0.871827452	1.020437693	0.30125095
S-VLDL-TG	Triglycerides	0.159187554	0.059124374	0.994830949	0.91778246	1.078347713	0.953044696
XS-VLDL-P	Lipoprotein particle	5.52E-05	1.36E-05	1.016407022	0.940713682	1.098190932	0.822052514
XS-VLDL-L	Total lipids	0.360495875	0.08756203	1.024779763	0.948320336	1.10740382	0.709832857
XS-VLDL-PL	Phospholipids	0.105470775	0.025990852	1.046326899	0.969202603	1.12958836	0.44593789
XS-VLDL-C	Cholesterol	0.185677905	0.049178562	0.992390178	0.916961408	1.074023681	0.913984006
XS-VLDL-CE	Esterified cholesterol	0.127548207	0.035114272	0.981604441	0.90643529	1.063007243	0.812628457
XS-VLDL-FC	Free cholesterol	0.058129713	0.014671642	1.017938788	0.941848408	1.100176384	0.812929397
XS-VLDL-TG	Triglycerides	0.069347194	0.021022802	1.064310682	0.986371859	1.148407893	0.234427151
IDL-P	Lipoprotein particle	0.000309501	7.63E-05	0.932851248	0.862812407	1.008575496	0.192779611
IDL-L	Total lipids	1.221760428	0.287741634	0.918931998	0.847840841	0.995984122	0.133489723
IDL-PL	Phospholipids	0.290776568	0.065822453	0.939978919	0.867246005	1.01881169	0.281640852
IDL-C	Cholesterol	0.831434301	0.213685083	0.901688882	0.831752896	0.977505272	0.055323323
**IDL-CE**	**Esterified cholesterol**	**0.612487887**	**0.158674775**	**0.898312844**	**0.828572559**	**0.973923113**	**0.045856408**
IDL-FC	Free cholesterol	0.218946415	0.056199615	0.913289953	0.84265261	0.989848637	0.105179388
IDL-TG	Triglycerides	0.099549496	0.025817887	1.06616638	0.988496155	1.149939475	0.216461569
L-LDL-P	Lipoprotein particle	0.000729563	0.000174549	0.920539061	0.850553525	0.996283171	0.133489723
**L-LDL-L**	**Total lipids**	**1.563533914**	**0.373933654**	**0.883143821**	**0.814987726**	**0.9569997**	**0.018247737**
**L-LDL-PL**	**Phospholipids**	**0.353300129**	**0.081978506**	**0.884869267**	**0.817127311**	**0.958227205**	**0.018674068**
**L-LDL-C**	**Cholesterol**	**1.113710125**	**0.280520877**	**0.874446284**	**0.806650734**	**0.94793976**	**0.01469116**
**L-LDL-CE**	**Esterified cholesterol**	**0.822251032**	**0.205717595**	**0.875257804**	**0.807429055**	**0.948784562**	**0.01469116**
**L-LDL-FC**	**Free cholesterol**	**0.291459177**	**0.076808692**	**0.875333675**	**0.807414816**	**0.948965795**	**0.01469116**
L-LDL-TG	Triglycerides	0.096523659	0.025364478	1.039886008	0.962895306	1.123032694	0.531159321
M-LDL-P	Lipoprotein particle	0.000293387	7.48E-05	0.910409235	0.841023606	0.985519276	0.080660592
**M-LDL-L**	**Total lipids**	**0.613620647**	**0.162866587**	**0.881775503**	**0.814100194**	**0.95507659**	**0.018247737**
**M-LDL-PL**	**Phospholipids**	**0.159624506**	**0.041381505**	**0.880359811**	**0.812780982**	**0.953557494**	**0.018247737**
**M-LDL-C**	**Cholesterol**	**0.421365132**	**0.115832283**	**0.876106566**	**0.808962427**	**0.948823689**	**0.01469116**
**M-LDL-CE**	**Esterified cholesterol**	**0.303812794**	**0.086140502**	**0.882714022**	**0.814846146**	**0.956234558**	**0.018247737**
**M-LDL-FC**	**Free cholesterol**	**0.117552351**	**0.031898277**	**0.864513822**	**0.798281825**	**0.936240969**	**0.008186471**
M-LDL-TG	Triglycerides	0.032630979	0.010260008	1.013786539	0.937386094	1.096413905	0.837364186
S-LDL-P	Lipoprotein particle	0.000169359	3.70E-05	0.929460294	0.85920771	1.005457037	0.180364815
**S-LDL-L**	**Total lipids**	**0.282184829**	**0.06442555**	**0.892892074**	**0.824972455**	**0.966403485**	**0.029604273**
**S-LDL-PL**	**Phospholipids**	**0.087373393**	**0.018417364**	**0.90079377**	**0.83294912**	**0.974164443**	**0.045545325**
**S-LDL-C**	**Cholesterol**	**0.179309598**	**0.043703605**	**0.884611325**	**0.817249953**	**0.957524921**	**0.018247737**
**S-LDL-CE**	**Esterified cholesterol**	**0.130429485**	**0.032673343**	**0.893956394**	**0.825574358**	**0.96800249**	**0.031695148**
**S-LDL-FC**	**Free cholesterol**	**0.048880103**	**0.012377815**	**0.871531033**	**0.806017999**	**0.942368957**	**0.011503208**
S-LDL-TG	Triglycerides	0.01550182	0.005654934	1.00033704	0.923923027	1.083070954	0.993368192
XL-HDL-P	Lipoprotein particle	0.000227885	9.07E-05	1.01343425	0.923890468	1.111656647	0.868470079
XL-HDL-L	Total lipids	0.164542315	0.075943645	1.032218947	0.941162208	1.132085357	0.67579479
XL-HDL-PL	Phospholipids	0.077162687	0.042520646	1.046378076	0.954541916	1.147049763	0.54175293
XL-HDL-C	Cholesterol	0.080282274	0.03320513	1.010320694	0.920530075	1.108869697	0.904733288
XL-HDL-CE	Esterified cholesterol	0.056964435	0.026548839	1.002533134	0.912335995	1.101647519	0.978582848
XL-HDL-FC	Free cholesterol	0.023317831	0.006938198	1.036442356	0.949180812	1.131726161	0.605869051

Metabolites	Class	Mean	sd	HR	HR_lower	HR_upper	FDR_P_value
XL-HDL-FC	Free cholesterol	0.023317831	0.006938198	1.036442356	0.949180812	1.131726161	0.605869051
L-HDL-P	Lipoprotein particle	0.001352123	0.000733498	0.98678947	0.896376132	1.086322385	0.87073602
L-HDL-L	Total lipids	0.629260499	0.308457392	0.993352482	0.902940255	1.092817766	0.95088643
L-HDL-PL	Phospholipids	0.313492302	0.14513919	0.997035818	0.907185661	1.095784981	0.978582848
L-HDL-C	Cholesterol	0.285677567	0.160014932	0.984813511	0.894495327	1.084251222	0.850326057
L-HDL-CE	Esterified cholesterol	0.220674012	0.124456233	0.981331604	0.891231054	1.080541026	0.822052514
L-HDL-FC	Free cholesterol	0.065003561	0.035892725	0.997102375	0.906423437	1.096852867	0.978582848
L-HDL-TG	Triglycerides	0.030090617	0.01196337	1.047922568	0.969177642	1.133065456	0.445383878
M-HDL-P	Lipoprotein particle	0.003776201	0.000897499	0.926493388	0.848297659	1.011897167	0.20654899
M-HDL-L	Total lipids	1.015896303	0.218062491	0.936438698	0.858671504	1.021249022	0.289441889
M-HDL-PL	Phospholipids	0.478524357	0.097637114	0.947084047	0.869621935	1.031446145	0.412832513
M-HDL-C	Cholesterol	0.483067252	0.117981482	0.918886776	0.840696997	1.004348666	0.174601196
M-HDL-CE	Esterified cholesterol	0.398252097	0.094688666	0.918004702	0.84023445	1.002973198	0.169818718
M-HDL-FC	Free cholesterol	0.084815155	0.023838425	0.925381168	0.84580596	1.01244298	0.20654899
M-HDL-TG	Triglycerides	0.054304699	0.018730971	1.030534114	0.9533429	1.113975424	0.623299649
**S-HDL-P**	**Lipoprotein particle**	**0.009562036**	**0.001275138**	**0.852737752**	**0.785886231**	**0.925276006**	**0.003747296**
**S-HDL-L**	**Total lipids**	**1.146534437**	**0.154727524**	**0.888741341**	**0.81901378**	**0.964405228**	**0.029604273**
S-HDL-PL	Phospholipids	0.654984966	0.091335545	0.906190544	0.835110302	0.983320765	0.078429719
**S-HDL-C**	**Cholesterol**	**0.438353023**	**0.059520943**	**0.852331058**	**0.785483528**	**0.924867559**	**0.003747296**
**S-HDL-CE**	**Esterified cholesterol**	**0.325125943**	**0.045966922**	**0.847437733**	**0.781442873**	**0.919006029**	**0.002995333**
**S-HDL-FC**	**Free cholesterol**	**0.113227087**	**0.01585317**	**0.889049747**	**0.818694465**	**0.965451076**	**0.029604273**
S-HDL-TG	Triglycerides	0.053196397	0.017221942	1.016112143	0.936465415	1.102532855	0.822052514

*** Total C, total cholesterol; Non HDL-C, non HDL cholesterol; Remnant-C, total cholesterol - non HDL cholesterol; VLDL-C, low density lipoprotein cholesterol; LDL-C, low density lipoprotein cholesterol; Clinical LDL-C, clinical measurement of low density lipoprotein cholesterol; HDL-C, high-density lipoprotein cholesterol; Total TG, total triglyceride; VLDL-TG, Low Density Lipoprotein Cholesterol Triester; LDL-TG, Low Density Lipoprotein Cholesterol Triester; HDL-TG, high-density lipoprotein cholesterol triglyceride; Total FC, total cholesterol ester; VLDL-FC, low density lipoprotein cholesterol ester; LDL-FC, low density lipoprotein cholesterol ester; HDL-FC, high-density lipoprotein cholesterol ester; Total CE, total cholesterol ester; VLDL-CE, low density lipoprotein cholesterol ester; LDL-CE, low density lipoprotein cholesterol ester; HDL-CE, high-density lipoprotein cholesterol ester; Total L, total lipoprotein; VLDL-L, Low Density Lipoprotein; LDL-L, low density lipoprotein; HDL-L, high-density lipoprotein; Total PL, total phospholipid content; VLDL-PL, low density lipoprotein phospholipid; LDL-PL, low density lipoprotein phospholipid; HDL-PL, high-density lipoprotein phospholipid; Total P, total amount of lipoprotein particles; VLDL-P, low density lipoprotein particles; LDL-P, low density lipoprotein particles; HDL-P, high-density lipoprotein particles; Phosphoglycerides, phosphoglycerides; TG/PG, triglyceride/phosphoglyceride ratio; Total choline; Phosphatidylcholine, phosphatidylcholine; Sphingomyelins, sphingomyelin; VLDL particle size, triglyceride low density lipoprotein particle size; LDL particle size, low density lipoprotein particle size; HDL particle size, high-density lipoprotein particle size; ApoB, Apolipoprotein B; ApoA1, Apolipoprotein A1; ApoB/ApoA1, apolipoprotein B/A1 ratio; Glucose; Lactate, lactic acid; Pyruvate, pyruvate; Citrate, citric acid; Total fatty acids; Unsaturation degree; Omega-3, Omega-3; Omega-6, Omega-6; PUFA, polyunsaturated fatty acid; MUFA, monounsaturated fatty acid; SFA, saturated fatty acid; LA, linolenic acid; DHA, docosahexaenoic acid; PUFA/MUFA, ratio of polyunsaturated fatty acids to monounsaturated fatty acids; Omega-6/Omega-3, Omega-6/Omega-3 ratio; Alanine; Glutamine; Glycine; Histidine, histidine; Total BCAA, total amount of branched chain amino acids; Isoleucine; Leucine, leucine; Valine, valine; Phenylalanine, phenylalanine; Tyrosine, tyrosine; 3-Hydroxybutyrate, 3-hydroxybutyric acid; Acetate, acetic acid; Acetoacetate, ethyl acetate; Acetone, acetone; Creatinine, creatinine; Albumin, albumin; Glycoprotein acetyls, acetyl glycoproteins; S. Small; 40. Very large; XS, very small; XXL, especially large.% Represent the percentage of total fatty acids in the fatty acid category, such as Omega-3%: The percentage of ω- fatty acids in total fatty acids.% In the lipoprotein category, it represents the percentage of a certain lipid in the total lipid in the lipoprotein subclass, such as L-HDL-C%: the percentage of cholesterol in the total lipid in large HDL.

Table S2 Codes used in the UK Biobank study to identify RA cases.

Source	UK Biobank data fields	Corresponding codes
**Hospital inpatient data**<br>RA dignosis of ICD-10, ICD-9	41720: “Diagnoses - ICD10”<br>41271: “Diagnoses - ICD9”	**ICD-9:** 71400, 71401, 71403, 71404, 71405, 71406, 71409<br>**ICD-10:** M05.0, M05.1, M05.2, M05.3, M05.8, M05.9, M06.0, M06.1, M06.2, M06.3, M06.4, M06.8, M06.9
**Medical conditions**<br>self-report past and current RA through a verbal interview by a trained nurse.	20002: “Non-cancer illness code, self-reported”	1464
**Prescription medication**<br>self-report past and current RA-relevant treatment medicine taken through a verbal interview by a trained nurse, **including the following medicine:**<br>1) Steroids: corticosteroids, depomedrone, triamcinolone, methylprednisolone, prednisolone, prednisone (also listed in UK Biobank as Deltacortril enteric; Deltastab; Precortisyl; Prednesola)<br>2) Synthetic DMARDs: Auranofin (also listed in UK Biobank as Ridaura), Azathioprine (also listed in UK Biobank as Imuran), hydroxychloroquine as (also listed in UK Biobank as Plaquenil),leflunomide (also listed in UK Biobank as Arava), methotrexate, methotrexate injections,myocrisin, penicillamine, sulfasalazine (also listed in UK Biobank as Sulazine, salazopyrin, sulphasalazine)<br>3) Biologic DMARDS: abatacept, adalimumab (also listed in UK Biobank as Humira injection solution), certolizumab, etanercept, golimumab, infliximab, rituximab, tocilizumab	20003: “Treatment/medication code”	1140874936,1140874940,1140874944,1140874950, 1140874954,1140874956,1140874978,1140868426, 1140883058,1140883060,1140883062,1140883064, 1140874976,1140800000,1140874930,1140868364, 1140868370,1140874936,1140874940,1140875316, 1140874944,1140874950,1140875400,1140875404, 1140909864,1140869930,1141145996,1140884308, 1140875392,1141166294,1141166302,1141166304, 1141166306,1140910036,1140869848,1140853054, 1140853056,1140875304,1140875306,1140875308, 1140909702,1141188900, 1140865670,1140865668, 1141188588,1141188594

Table S3 Tests for weak instrument and statistical power in the two-sample MR analyses.

Metabolites	Class	nSNPs used as IVs	*F* statistics for IVs (mean, min-max)	Heterogeneity test for IVs			
				Cochrane's Q	*P* value	Rucker's Q	*P value*
Albumin	Fluid balance	29	92.6 (30.4-840.8)	65.8	1.60e-05	62.5	2.78E-05
GlycA	Glycoprotein acetyls	61	96.4(26.6-986.2)	97.9	2.36E-06	95.1	3.47E-06
Omega-3%	Fatty acids	41	289.7(26.5-8571.6)	27.8	0.419061801	63.6	0.000742174
Omega-3	Fatty acids	52	234.1(26.2-6315.3)	54.2	0.066613961	65.1	0.000486077
DHA%	Fatty acids	28	228.4(29.2-4288)	17.2	0.700351738	83.6	1.85E-05
DHA	Fatty acids	48	183.4(29.7-4825.6)	59	0.009253712	69.5	0.006360792
PUFA	Fatty acids	66	128.6(25.8-700)	955	3.18E-168	69	0.003986226
Valine	Amino acids	22	113(29.8-673)	13.6	0.480911944952683	27.7	0.374293935
Citrate	Glycolysis	38	121.7(28.8-684.7)	50.5	0.005655625	62.2	0.022987791
S-HDL-CE	Lipoprotein lipids in subclasses	50	116.7(29.4-1328)	63.7	0.001058139	55.6	0.063250078
S-HDL-P	Lipoprotein lipids in subclasses	49	106.5(28.5-1254.2)	65.1	0.000716753	102.5	3.48E-06
S-HDL-C	Lipoprotein lipids in subclasses	54	95.7(27.8-1142.2)	84.9	1.91E-05	212.9	1.04E-24
S-HDL-L	Lipoprotein lipids in subclasses	48	112.5(29-1074.7)	937.1	1.55E-170	58.3	0.008021925
S-HDL-FC	Lipoprotein lipids in subclasses	52	102.2(29.2-547.9)	72	0.002685761	45.2	0.015619944
S-LDL-L	Lipoprotein lipids in subclasses	53	198.7(27.7-4788)	219.8	1.45E-25	58.7	0.028214042
S-LDL-CE	Lipoprotein lipids in subclasses	55	159.8(26.4-3075.5)	497.6	3.86E-78	214.5	5.46E-25
S-LDL-PL	Lipoprotein lipids in subclasses	55	247.2(25.3-7256.6)	218.4	1.32E-24	207.6	8.81E-24
S-LDL-FC	Lipoprotein lipids in subclasses	55	247.1(28.8-7648.3)	70.4	0.003947629	503.5	9.06E-79
S-LDL-C	Lipoprotein lipids in subclasses	55	180.9(25.6-4423.8)	225.9	2.75E-26	214.8	1.10E-24
M-LDL-FC	Lipoprotein lipids in subclasses	53	206.5(27.2-5655.7)	71.1	0.005986024	90.2	1.49E-05
M-LDL-C	Lipoprotein lipids in subclasses	51	171.2(28.2-3598.4)	218.9	2.08E-25	52.8	0.068611533
M-LDL-L	Lipoprotein lipids in subclasses	51	174.1(28.2-3578.7)	223.5	3.16E-26	951.9	3.13E-168
M-LDL-PL	Lipoprotein lipids in subclasses	51	195.1(27.4-4420.6)	214.1	1.44E-24	205	2.58E-23
M-LDL-CE	Lipoprotein lipids in subclasses	54	150.9(26.8-2800.2)	505.4	1.26E-78	931.8	3.92E-170
L-LDL-C	Lipoprotein lipids in subclasses	55	186.4(26.7-4787.4)	62.4	0.027897409	68.6	0.004403767
L-LDL-CE	Lipoprotein lipids in subclasses	53	185.9(27.4-4451.7)	56.3	0.069591964	493.3	8.29E-78
L-LDL-FC	Lipoprotein lipids in subclasses	58	192.7(29.9-5472.3)	105.6	2.17E-06	16.6	0.680688276
L-LDL-L	Lipoprotein lipids in subclasses	53	190.5(27.5-4566.2)	58.8	0.035233354	208.8	2.79E-23
L-LDL-PL	Lipoprotein lipids in subclasses	51	196.6(29.7-4794.9)	92.4	1.20E-05	130.3	7.20E-09
IDL-CE	Lipoprotein lipids in subclasses	67	175.9(26.7-5549.7)	132	6.84E-09	62.5	2.78E-05

Table S4 Tests for weak instrument and statistical power in the one-sample MR analyse.

Metabolites	Class	*F* statistics for IVs	Minimum detectable OR(at 80% power)
Albumin	Fluid balance	95.3	0.20/1.83
GlycA	Glycoprotein acetyls	95.5	0.42/1.58
Omega-3%	Fatty acids	244.9	0.54/1.47
Omega-3	Fatty acids	217.9	0.56/1.43
DHA%	Fatty acids	184.4	0.38/1.63
DHA	Fatty acids	147.5	0.46/1.55
PUFA	Fatty acids	132	0.23/1.77
Valine	Amino acids	83.2	0.23/1.77
Citrate	Glycolysis	120.5	0.33/1.66
S-HDL-CE	Lipoprotein lipids in subclasses	119.1	0.49/1.50
S-HDL-P	Lipoprotein lipids in subclasses	107.9	0.48/1.53
S-HDL-C	Lipoprotein lipids in subclasses	95.6	0.44/1.56
S-HDL-L	Lipoprotein lipids in subclasses	114.6	0.53/1.48
S-HDL-FC	Lipoprotein lipids in subclasses	102.2	0.49/1.50
S-LDL-L	Lipoprotein lipids in subclasses	189.8	0.52/1.44
S-LDL-CE	Lipoprotein lipids in subclasses	169.5	0.37/1.62
S-LDL-PL	Lipoprotein lipids in subclasses	239.2	0.37/1.63
S-LDL-FC	Lipoprotein lipids in subclasses	243.8	0.49/1.50
S-LDL-C	Lipoprotein lipids in subclasses	172.7	0.53/1.48
M-LDL-FC	Lipoprotein lipids in subclasses	197.4	0.47/1.53
M-LDL-C	Lipoprotein lipids in subclasses	162.9	0.50/1.50
M-LDL-L	Lipoprotein lipids in subclasses	165	0.74/1.27
M-LDL-PL	Lipoprotein lipids in subclasses	185.2	0.72/1.30
M-LDL-CE	Lipoprotein lipids in subclasses	142.6	0.66/1.34
L-LDL-C	Lipoprotein lipids in subclasses	176.3	0.58/1.42
L-LDL-CE	Lipoprotein lipids in subclasses	176.2	0.58/1.43
L-LDL-FC	Lipoprotein lipids in subclasses	187.1	0.63/1.37
L-LDL-L	Lipoprotein lipids in subclasses	180.9	0.45/1.55
L-LDL-PL	Lipoprotein lipids in subclasses	185.6	0.42/1.58
**IDL-CE**	**Lipoprotein lipids in subclasses**	**166.2**	**0.68/1.32**

Table S5. Non-MHC region SNPs used to construct PRS

RSID	Chr	Position	Ref	Alt	Nearest Gene	Region
rs60733400	1	2516781	G	C	*LOC100996583*	downstream
rs2240336	1	17674402	C	T	*PADI4*	intronic
rs28411352	1	38278579	C	T	*MTF1*	UTR3
rs773566	1	113821584	T	C	*LOC643441;MAGI3*	intergenic
rs72685677	1	113840826	C	T	*LOC643441;MAGI3*	intergenic
rs12061333	1	113860779	C	A	*LOC643441;MAGI3*	intergenic
rs61819363	1	114024462	C	T	*MAGI3*	intronic
rs66782936	1	114087710	T	G	*MAGI3*	intronic
rs2884704	1	114114717	T	C	*MAGI3*	intronic
rs2476601	1	114377568	A	G	*PTPN22*	exonic
rs2476602	1	114396955	G	A	*AP4B1-AS1*	ncRNA_intronic
rs1217390	1	114451307	C	T	*DCLRE1B*	intronic
rs1503836	1	114541498	G	T	*OLFML3;SYT6*	intergenic
rs34382796	1	114564892	G	A	*OLFML3;SYT6*	intergenic
rs624988	1	117263790	T	C	*C1orf137;CD2*	intergenic
rs61828284	1	173299743	C	T	*LOC100506023*	ncRNA_intronic
rs2105325	1	173349725	A	C	*LOC100506023*	ncRNA_intronic
rs34695944	2	61124850	T	C	*REL*	intronic
rs1858036	2	65598241	A	G	*SPRED2*	intronic
rs9653442	2	100825367	C	T	*LINC01104*	ncRNA_intronic
rs13426947	2	191933254	G	A	*STAT4*	intronic
rs231389	2	204634730	C	T	*CD28;CTLA4*	intergenic
rs3087243	2	204738919	G	A	*CTLA4*	downstream
rs4416363	3	17025606	A	G	*PLCL2*	intronic
rs9310852	3	27784997	A	T	*EOMES;LINC01980*	intergenic
rs73081554	3	58302935	C	T	*HTD2;RPP14*	intronic
rs34046593	4	26111593	G	A	*SMIM20;RBPJ*	intergenic
rs7731626	5	55444683	G	A	*ANKRD55*	intronic
rs2561477	5	102608924	G	A	*C5orf30*	intronic
rs2234067	6	36355654	A	T	*ETV7*	upstream
rs2233434	6	44232920	A	G	*NFKBIE*	exonic
rs17264332	6	138005515	A	T	*LOC102723649;LINC02539*	intergenic
rs61117627	6	138243700	G	A	*TNFAIP3;LINC02528*	intergenic
rs2451258	6	159506600	C	T	*TAGAP;LOC101929122*	intergenic
rs1571878	6	167540842	C	T	*CCR6*	intronic
rs12539741	7	128596805	C	T	*TNPO3*	intronic
rs11574914	9	34710338	G	A	*CCL21*	upstream
rs10985070	9	123636121	C	A	*PHF19*	intronic
rs706778	10	6098949	C	T	*IL2RA*	intronic
rs10796038	10	6397964	A	G	*LINC02656;PRKCQ*	intergenic
rs537544	10	8108382	C	T	*GATA3*	intronic
rs12764378	10	63800004	G	A	*ARID5B*	intronic
rs10790268	11	118729391	A	T	*DDX6;CXCR5*	intergenic
rs4936059	11	128502496	A	G	*ETS1;LOC101929538*	intergenic
rs9603608	13	40318819	A	C	*COG6*	intronic
rs8032939	15	38834033	T	C	*RASGRP1*	intronic
rs8026898	15	69991417	G	A	*PCAT29;LINC00593*	intergenic
rs71411040	15	70001013	T	C	*PCAT29;LINC00593*	intergenic
rs2139493	16	86021624	T	C	*IRF8;LINC01082*	intergenic
rs12232497	17	38040119	T	C	*ZPBP2;GSDMB*	intergenic
rs592390	18	12822314	T	C	*PTPN2*	intronic
rs62097857	18	12857758	G	A	*PTPN2*	intronic
rs2278442	19	10444826	G	A	*ICAM3*	splicing
rs11085727	19	10466123	C	T	*TYK2*	intronic
rs4239702	20	44749251	T	C	*CD40*	intronic
rs8133843	21	36738242	G	A	*RUNX1;LOC100506403*	intergenic
rs1893592	21	43855067	A	G	*UBASH3A*	splicing
rs2069235	22	39747780	G	T	*SYNGR1*	intronic

Table S6. Effect estimates for the five amino acids of haplotypes of *HLA-DRB1*, *HLA*-B and *HLA-DPB1* associated with RA Risk.

**HLA-DRβ1 Amino Acid Position**				**Multivariate Odds Ratio (95% Confidence Interval)**		**Unadjusted Allele Frequency**		**Classical *HLA-DRB1* Alleles**
**11**	**13**	**71**	**74**			**Controls**	**Cases**	
V	H	K	A	4.44	(4.02 – 4.91)	0.106	0.316	***04:01**
V	H	R	A	4.22	(3.75 – 4.75)	0.056	0.141	***04:08, *04:05, *04:04**
	F							***10:01**
L	F	R	A	2.17	(1.94 – 2.42)	0.109	0.143	***01:02, *01:01**
P	R	R	A	2.04	(1.59 – 2.62)	0.013	0.012	*16:01
V	H	R	E	1.65	(1.24 – 2.19)	0.01	0.009	*04:03, *04:07
D	F	R	E	1.65	(1.29 – 2.10)	0.011	0.013	*09:01
V	H	E	A	1.43	(1.04 – 1.96)	0.011	0.006	*04:02
S	S	K	A	1.04	(0.76 – 1.41)	0.012	0.006	*13:03
P	R	A	A	1	REF	0.142	0.092	*15:01, *15:02
G	Y	R	Q	0.91	(0.80 – 1.03)	0.133	0.064	*07:01
S	S	R	A	0.88	(0.77 – 1.00)	0.103	0.049	*11:01, *11:04
	G							*12:01
S	S	R	E	0.84	(0.67 – 1.05)	0.025	0.012	*14:01
L	F	E	A	0.73	(0.42 – 1.27)	0.004	0.002	*01:03
S	G	R	L	0.71	(0.57 – 0.89)	0.028	0.013	*08:01, *08:04
S	S	K	R	0.63	(0.54 – 0.73)	0.128	0.083	*03:01
S	S	E	A	0.59	(0.51 – 0.68)	0.112	0.041	*11:02, *11:03, *13:01, *13:02
**HLA-B Amino Acid Position 9**								**Classical *HLA-B* Allele**
D				2.12	(1.89 – 2.38)	0.118	0.13	*08
H,Y				1	REF	0.882	0.87	*07, *13, *14, *15, *18, *27, *35, *37, *38, *39, *40, *41, *44, *45, *47, *49, *50, *51, *52, *53, *55, *56, *57, *58, *73
**HLA-DPβ1 Amino Acid Position 9**								**Classical *HLA-DPB1* Alleles**
F				1.4	(1.31 – 1.50)	0.728	0.799	*02:01, *02:02, *04:01, *04:02, *05:01, *16:01, *19:01, *23:01
H,Y				1	REF	0.272	0.201	*01:01, *03:01, *06:01, *09:01, *10:01, *11:01, *13:01, *14:01, *15:01, *17:01, *20:01

Effect Estimates for the Five Amino Acids Associated with RA Risk.
Estimated effects for haplotypes of HLA-DRB1, HLA-B and HLA-DPB1.
Classical alleles of HLA-DRB1 are grouped based on amino acid residues at positions 11 (or 13), 71, and 74 within DRβ1. We have bolded the classical shared epitope alleles. For each haplotype, the multivariate effect is given as an odds ratio, taking the most frequent haplotype (PRAA) in the control samples as the reference (that is, odds ratio = 1). All effects are conditional on Asp-9 in HLA-B and Phe-9 in HLA-DPβ1. Unadjusted haplotype frequencies are given for cases and controls. HLA-DRB1 haplotypes in aggregate explain 9.7% of the phenotypic variance of rheumatoid arthritis. The multivariate effect sizes, allele frequencies, and classical alleles corresponding to Asp-9 in HLA-B and Phe-9 in HLA-DPβ1 are also listed.

Table S7 The weighted coefficients of metabolic risk score based the 37 clinically validated biomarkers.

Class	Metabolic biomarker (SD units)	β-weight	Included in MRS
Cholesterols	Total cholesterol	-	NO
	VLDL cholesterol	0.02590271	YES
	LDL cholesterol	-0.1970568	YES
	HDL cholesterol	0.03543448	YES
Triglycerides	Total Triglycerides	-0.2656116	YES
Fatty acids	Total fatty acids	-	NO
	Omega-3 fatty acids	-0.06095256	YES
	Omega-6 fatty acids	-	NO
	Polyunsaturated fatty acids	-	NO
	Monounsaturated fatty acids	-	NO
	Saturated fatty acids	0.2476603	YES
	Docosahexenoic acid	-	NO
Fatty acid ratios	Ratio of omega-3 fatty acids to total fatty acids	-	NO
	Ratio of omega-6 fatty acids to total fatty acids	-	NO
	Ratio of polyunsaturated fatty acids to total fatty acids	-	NO
	Ratio of monounsaturated fatty acids to total fatty acids	0.02585942	YES
	Ratio of saturated fatty acids to total fatty acids	-	NO
	Ratio of docosahexaenoic acid to total fatty acids	-	NO
	Ratio of polyunsaturated fatty acids to monounsaturated fatty acids	-	NO
	Ratio of omega-6 fatty acids to omega-3 fatty acids	-	NO
Apolipoproteins	Apolipoprotein B	-	NO
	Apolipoprotein A	-	NO
	Ratio of apolipoprotein B ratio to apolipoprotein A1	-	NO
Amino acids	Alanine	-0.05154659	YES
	Glycine	0.001461825	YES
	Histidine	-0.05582636	YES
Branched-chain amino acids	Isoleucine	0.1670207	YES
	Leucine	-	NO
	Valine	-0.3383579	YES
	Total concentration of branched-chain amino acids (leucine, isoleucine, valine)	-	NO
Aromatic amino acids	Phenylalanine	0.02775649	YES
	Tyrosine	0.02775649	YES
Glycolysis related metabolites	Glucose	-0.02527383	YES
	Lactate	-	NO
Fluid balance	Creatinine	-	NO
	Albumin	-0.3111579	YES
Inflammation	Glycoprotein acetyls (GlycA)	0.4311847	YES

Table S8 The weighted coefficients of metabolic risk score based the 143 NMR-quantified metabolites

Class	Metabolic biomarker (SD units)	β-weight	Included in MRS
Fluid balance	Albumin	-0.279116386	YES
Inflammation	Glycoprotein acetyls	0.371169131	YES
Lipoprotein particle size	Average diameter for VLDL particles	-0.156626335	YES
Fatty acid ratios	Saturated fatty acids to total fatty acids percentage	0.017599088	YES
Amino acids	Histidine	0.005266765	YES
Lipoprotein lipids	Phospholipids in small LDL	-0.073334270	YES

Table S9 The basic characteristics of participants with and without metabolic biomarker profiling.

Characteristics	Current study population (n=93,800)	Excluded population (n=24,211)
Age at blood collection (years)	56.8 (8.0)	54.5 (8.4)
Sex, n (%)		
Women	49,562 (52.8)	13,163 (54.4)
Men	44,238 (47.2)	11,048 (45.6)
Education level, n (%)		
University degree	29,008 (30.9)	10,141 (41.9)
No university degree	64,792 (69.1)	13,217 (54.6)
Smoking status, n (%)		
Never	51,087 (54.5)	13,211 (54.6)
Previous	33,085 (35.3)	7,533 (31.1)
Current	9,628 (10.3)	3,086 (12.7)
Physical activity (MET minutes/week)	2,655.3 (2,744.3)	2489.9 (2686.1)
Body mass index (kg/m^2)	27.5 (4.7)	27.6 (5.0)
Fasting time (hours)	3.7 (2.4)	4.1 (2.7)
Clinical blood chemistry measures		
Total cholesterol (mmol/L)	5.72 (1.14)	5.53 (1.13)
HDL_cholesterol (mmol/L)	1.44 (0.37)	1.39 (0.36)
LDL_cholesterol (mmol/L)	3.59 (0.87)	3.47 (0.86)
Triglycerides (mmol/L)	1.78 (1.01)	1.72 (1.04)
Albumin (g/L)	45.24 (2.61)	45.05 (2.76)
C-reactive protein (mg/L)	2.56 (4.24)	2.90 (5.01)
Rheumatoid factor (IU/ml)	23.80 (19.24)	30.25 (24.8)
Prevalent diseases, n (%)		
CVD	27,961 (29.8)	7,220 (29.8)
Diabetes	4,554 (4.9)	2,036 (8.4)
Cancers	7,254 (7.7)	1513 (6.3)

Table S10 Cross-trait genetic correlation between candidate metabolic biomarkers and RA.

	RA	Albumin	GlycA	PUFA	Omega-3%	Omega-3	DHA%	DHA	Citrate	Valine	S-LDL-PL	S-LDL-L	S-LDL-FC	S-LDL-CE	S-LDL-CE	S-LDL-C	S-HDL-P	S-HDL-L	S-HDL-FC	S-HDL-CE	S-HDL-C	M-LDL-PL	M-LDL-L	M-LDL-FC	M-LDL-CE	M-LDL-C	L-LDL-PL	L-LDL-L	L-LDL-FC	L-LDL-CE	L-LDL-C
RA		1.01E-05	0.082335	0.059848	0.0049213	0.0043838	0.025439	0.0034536	0.71409	0.050186	0.39601	0.37382	0.55806	0.37894	0.40042	0.1153	0.15218	0.090809	0.1247	0.10069	0.44319	0.36794	0.44566	0.3104	0.33407	0.59683	0.40176	0.54523	0.2559	0.3039	0.76402
Albumin	-0.1917		0.0047569	0.0063625	0.00016894	0.00045203	0.00010232	2.13E-08	0.98812	0.17347	0.058605	0.035169	0.10233	0.03055	0.030085	0.0044445	0.012655	0.00032019	0.0050643	0.0020367	0.025738	0.017345	0.017946	0.019738	0.014181	0.018313	0.0044415	0.014568	0.0017559	0.0021181	0.096769
GlycA	0.06	-0.186		1.92E-07	0.21014	9.60E-06	6.16E-21	0.12681	0.39308	6.88E-17	0.036269	0.00055474	0.11737	2.30E-06	0.00063936	6.84E-09	1.73E-13	2.28E-06	2.61E-05	7.19E-06	7.02E-05	7.21E-06	0.4753	1.73E-07	3.00E-05	0.29809	0.17991	0.003758	0.13257	0.69981	0.00047684
PUFA	-0.081	0.1501	0.3691		5.43E-12	4.01E-131	0.80222	3.83E-40	0.52015	0.2736	1.45E-17	2.25E-32	3.10E-06	2.79E-106	4.57E-34	4.56E-26	1.09E-20	9.34E-128	2.27E-14	1.17E-23	7.47E-40	4.54E-75	1.96E-25	2.37E-135	1.59E-66	1.25E-44	1.62E-40	2.15E-17	1.08E-45	1.73E-56	7.20E-15
Omega-3%	-0.1397	0.1976	-0.0729	0.4245		0	1.78E-94	0	0.0017753	0.17651	0.0071182	0.011828	0.013877	0.013852	0.011931	0.095151	0.12276	0.00039611	0.21107	0.061375	0.0097077	0.0083991	0.003478	0.014847	0.0084673	0.0010264	0.00086993	0.00040828	0.0005675	0.00018817	0.00013603
Omega-3	-0.1249	0.1943	0.291	0.8042	0.841		1.12E-05	0	0.018002	8.42E-05	8.56E-07	4.09E-08	0.0012027	1.14E-14	3.38E-08	2.82E-11	1.90E-14	3.27E-27	1.09E-06	9.22E-10	3.84E-09	1.15E-12	2.19E-07	7.23E-17	1.20E-11	2.01E-10	1.92E-09	4.76E-07	4.40E-10	3.06E-12	1.74E-06
DHA%	-0.0977	0.1865	-0.5085	-0.0171	0.7622	0.3409		3.99E-86	0.07128	7.16E-09	0.51524	0.038077	0.0016732	0.0005215	0.085797	0.0035002	2.45E-06	0.19177	0.22582	0.19578	0.025494	0.0045701	0.0025286	0.00017038	0.018064	0.0050595	0.011225	1.42E-06	0.015716	0.00062849	2.35E-07
DHA	-0.1335	0.2609	-0.0944	0.5992	0.9254	0.849	0.7658		0.049381	0.1897	4.24E-09	1.57E-08	1.21E-07	2.89E-07	2.26E-09	8.26E-07	1.20E-05	2.63E-17	1.87E-06	1.59E-08	3.03E-09	2.77E-09	3.26E-12	4.40E-07	2.81E-10	1.38E-17	3.43E-16	8.17E-21	3.93E-17	7.13E-21	1.70E-24
Citrate	-0.0197	-9.00E-04	-0.0536	0.039	-0.1921	-0.1438	-0.1004	-0.1144		0.0143	0.3119	0.4	0.3409	0.4954	0.4073	0.1089	0.0751	0.3848	0.0843	0.1149	0.7391	0.7436	0.419	0.8527	0.6914	0.3115	0.2548	0.161	0.251	0.217	0.1563
Valine	-0.0973	-0.0916	0.4323	0.0678	0.0741	0.2189	-0.2753	-0.0756	-0.1852		0.86049	0.1431	0.00144	0.018185	0.31216	0.022062	0.00021303	0.21644	0.26944	0.2373	0.088089	0.037302	0.014761	0.0048424	0.10518	0.0099791	0.0384	6.07E-07	0.067782	0.0040245	1.79E-08
S-LDL-PL	-0.0518	0.1278	0.1993	0.7232	0.2713	0.5272	0.0427	0.4584	0.0868	-0.013		0	1.02E-45	6.51E-114	0	6.27E-05	0.014629	1.51E-07	0.0028025	0.00013309	2.35E-131	3.30E-126	1.40E-80	2.06E-74	3.94E-129	4.17E-95	1.25E-190	4.45E-20	2.85E-213	1.01E-116	8.98E-09
S-LDL-L	-0.0495	0.1392	0.4176	0.8556	0.2568	0.6469	-0.1506	0.4245	0.0709	0.1271	0.9454		1.63E-09	0	0	4.46E-09	0.00047945	3.70E-11	2.96E-07	9.05E-09	0	0	7.93E-40	0	0	1.05E-54	7.02E-66	1.13E-07	4.39E-87	1.15E-53	0.00016516
S-LDL-FC	-0.039	0.1291	-0.1298	0.5132	0.2727	0.3132	0.3626	0.5224	0.0934	-0.2842	0.8591	0.7038		1.15E-05	8.16E-13	0.0062467	0.20518	0.00018772	0.0064801	0.0020374	4.51E-09	1.25E-07	0	3.92E-05	1.71E-09	9.51E-129	3.01E-95	0	6.18E-73	3.35E-144	1.49E-42
S-LDL-CE	-0.0433	0.1325	0.4914	0.8632	0.2141	0.6478	-0.246	0.3674	0.0498	0.1844	0.8732	0.986	0.5884		0	1.70E-17	2.69E-07	5.29E-23	6.72E-11	2.15E-14	0	0	4.97E-28	0	0	4.04E-38	4.59E-46	1.66E-05	1.24E-60	1.72E-35	0.0012404
S-LDL-CE	-0.0478	0.1454	0.3865	0.8618	0.2561	0.6348	-0.1183	0.4444	0.0699	0.0843	0.9376	0.9939	0.7403	0.9811		1.25E-09	0.000309	1.29E-11	2.72E-08	1.25E-09	0	0	2.08E-75	0	0	3.77E-103	6.99E-126	1.54E-11	2.32E-173	2.88E-96	2.64E-06
S-LDL-C	-0.0685	0.1671	0.3938	0.6093	0.1125	0.4445	-0.1565	0.3028	-0.1141	0.1423	0.324	0.5402	0.2713	0.6031	0.5815		0	0	0	0	2.67E-11	1.04E-16	4.11E-08	1.87E-24	1.95E-15	5.51E-11	3.19E-10	3.42E-06	3.86E-11	1.24E-11	0.0002251
S-HDL-P	-0.0609	0.1478	0.471	0.6724	0.1109	0.5034	-0.2414	0.2893	-0.126	0.2122	0.2907	0.5657	0.1548	0.6427	0.592	0.9838		0	0	0	3.94E-05	8.00E-07	0.00123	1.35E-09	2.37E-06	8.98E-05	0.00025818	0.01121	0.00013272	6.64E-05	0.019374
S-HDL-L	-0.0747	0.2026	0.3493	0.7969	0.2335	0.5975	-0.0723	0.4659	-0.055	0.0825	0.4771	0.6662	0.3876	0.7052	0.6978	0.9358	0.9522		0	0	2.93E-13	2.07E-20	1.66E-10	4.95E-31	9.57E-19	1.37E-15	8.73E-14	9.02E-12	6.57E-15	4.38E-18	3.57E-10
S-HDL-FC	-0.0687	0.1651	0.2902	0.4645	0.0782	0.3236	-0.0725	0.2701	-0.1274	0.0712	0.2058	0.3841	0.2559	0.441	0.439	0.9751	0.9265	0.8665		0	3.54E-10	2.21E-13	1.23E-07	1.64E-15	5.25E-13	5.70E-10	1.37E-09	6.43E-06	1.84E-10	2.46E-10	0.00039378
S-HDL-CE	-0.0729	0.1794	0.3147	0.5642	0.1214	0.4041	-0.0751	0.3281	-0.1124	0.0771	0.2842	0.4699	0.2978	0.5235	0.5199	0.9908	0.9582	0.9245	0.9913		1.91E-11	5.38E-16	1.34E-08	3.33E-20	3.12E-15	9.43E-12	4.30E-11	1.93E-07	4.77E-12	1.48E-12	1.63E-05
S-HDL-C	-0.0414	0.1434	0.4535	0.8435	0.2509	0.645	-0.1507	0.4295	0.0258	0.1459	0.8855	0.9706	0.6811	0.9745	0.9787	0.7056	0.7159	0.781	0.5715	0.643		0	1.64E-64	0	0	7.56E-73	2.77E-74	2.89E-08	3.96E-94	2.85E-59	0.00014053
M-LDL-PL	-0.0459	0.1471	0.4767	0.8585	0.2337	0.6507	-0.1926	0.4079	0.0242	0.17	0.8718	0.9761	0.6371	0.9868	0.9804	0.6931	0.7141	0.7771	0.5489	0.6246	0.9953		1.13E-50	0	0	3.47E-61	1.51E-67	2.72E-07	1.94E-88	1.22E-52	0.00026216
M-LDL-L	-0.0456	0.1624	0.0507	0.6997	0.2801	0.4602	0.2202	0.5524	0.0673	-0.1882	0.8796	0.8272	0.9465	0.7597	0.8689	0.5438	0.4521	0.6448	0.4986	0.5506	0.8481	0.8146		5.08E-30	3.55E-72	0	0	0	0	0	9.25E-49
M-LDL-FC	-0.0489	0.1407	0.523	0.8528	0.2046	0.6466	-0.2601	0.3596	0.0131	0.2189	0.8301	0.9643	0.5531	0.9894	0.9637	0.6855	0.7207	0.762	0.532	0.6078	0.979	0.9933	0.7483		0	1.30E-37	4.34E-43	4.27E-05	4.89E-57	1.80E-33	0.0029503
M-LDL-CE	-0.0512	0.1552	0.4401	0.8658	0.2395	0.6437	-0.1586	0.429	0.0303	0.1331	0.8808	0.975	0.6766	0.981	0.9855	0.6931	0.7044	0.7805	0.5563	0.6311	0.9927	0.997	0.8462	0.9872		8.09E-88	4.57E-97	1.31E-09	1.75E-131	1.98E-75	1.90E-05
M-LDL-C	-0.0287	0.1495	0.0742	0.7773	0.2895	0.5163	0.1789	0.5697	0.0792	-0.1899	0.8726	0.8425	0.9024	0.7882	0.8818	0.5458	0.489	0.691	0.4785	0.5472	0.8514	0.8281	0.9766	0.771	0.8589		0	0	0	0	3.85E-147
L-LDL-PL	-0.0461	0.1836	0.1097	0.8401	0.3326	0.5915	0.1595	0.6037	0.0962	-0.1579	0.897	0.8686	0.885	0.8185	0.9026	0.5684	0.5384	0.7344	0.4804	0.5599	0.8694	0.851	0.9691	0.7983	0.8785	0.9932		7.92E-260	0	0	2.24E-94
L-LDL-L	-0.0322	0.1597	-0.2023	0.6023	0.289	0.3517	0.3918	0.5838	0.1178	-0.3811	0.749	0.6286	0.9341	0.5382	0.683	0.3551	0.2576	0.5125	0.3449	0.3975	0.6233	0.5867	0.9262	0.5063	0.6347	0.9351	0.9126		8.78E-173	0	0
L-LDL-FC	-0.0629	0.2007	0.1264	0.8618	0.3415	0.6131	0.1475	0.6107	0.0953	-0.1382	0.8995	0.8825	0.871	0.837	0.9147	0.5861	0.558	0.7495	0.4941	0.5743	0.8822	0.8667	0.9635	0.8178	0.8929	0.985	0.9976	0.8957		0	2.70E-66
L-LDL-CE	-0.0552	0.1893	0.0285	0.7952	0.3204	0.5353	0.2198	0.6071	0.0951	-0.2112	0.876	0.8361	0.9076	0.7764	0.8735	0.5237	0.4638	0.6851	0.4566	0.5286	0.8343	0.8123	0.9737	0.7537	0.8444	0.9922	0.9952	0.9443	0.9921		9.40E-158
L-LDL-C	-0.0149	0.1048	-0.2341	0.612	0.3144	0.3717	0.4038	0.5966	0.1176	-0.402	0.6388	0.5278	0.8153	0.4526	0.5821	0.3079	0.2494	0.5048	0.2906	0.354	0.5159	0.4877	0.8184	0.4155	0.5358	0.882	0.8542	0.9596	0.8311	0.8853	

	rg, genetic correlation
	p value

Table S11 The one and two-sample MR analyses on causal relationship of candidate metabolic biomarkers with RA.

Metabolites	Class	Causal estimates using a two-stage method in the one-sample analyses		Causal estimates using inverse variance weighted (IVW) method in the two-sample analyses	
		HR (95% CI)	*p* value	OR (95% CI)	*p* value
Albumin	Fluid balance	1.57 (0.89-2.76)	0.12	0.94 (0.78-1.12)	0.48
GlycA	Glycoprotein acetyls	0.83 (0.55-1.25)	0.37	1.02 (0.92-1.13)	0.73
Omega-3%	Fatty acids	1.26 (0.91-1.75)	0.17	1.06 (0.98-1.16)	0.16
Omega-3	Fatty acids	1.20 (0.88-1.63)	0.24	1.04 (0.97-1.12)	0.27
DHA%	Fatty acids	1.11 (0.71-1.72)	0.65	1.11 (0.99-1.24)	0.05
DHA	Fatty acids	1.32 (0.91-1.93)	0.15	1.05 (0.95-1.18)	0.35
PUFA	Fatty acids	1.30 (0.93-1.82)	0.12	0.98 (0.91-1.07)	0.67
Valine	Amino acids	0.57 (0.27-1.19)	0.13	1.00 (0.87-1.17)	0.92
Citrate	Glycolysis	0.87 (0.52-1.33)	0.67	1.03 (0.91-1.17)	0.63
S-HDL-CE	Lipoprotein lipids in subclasses	0.73 (0.49-1.11)	0.14	0.99 (0.88-1.13)	0.93
S-HDL-P	Lipoprotein lipids in subclasses	0.76 (0.46-1.27)	0.3	0.96 (0.87-1.06)	0.45
S-HDL-C	Lipoprotein lipids in subclasses	1.37 (0.77-2.44)	0.28	0.98 (0.83-1.15)	0.77
S-HDL-L	Lipoprotein lipids in subclasses	0.78 (0.49-1.25)	0.3	1.04 (0.84-1.27)	0.73
S-HDL-FC	Lipoprotein lipids in subclasses	1.26 (0.82-1.93)	0.28	0.95 (0.85-1.06)	0.38
S-LDL-L	Lipoprotein lipids in subclasses	0.89 (0.65-1.21)	0.45	0.93 (0.80-1.07)	0.3
S-LDL-CE	Lipoprotein lipids in subclasses	0.94 (0.67-1.33)	0.74	0.90 (0.78-1.04)	0.17
S-LDL-PL	Lipoprotein lipids in subclasses	0.92 (0.70-1.21)	0.53	0.95 (0.83-1.10)	0.5
S-LDL-FC	Lipoprotein lipids in subclasses	0.89 (0.68-1.17)	0.41	1.02 (0.94-1.11)	0.63
S-LDL-C	Lipoprotein lipids in subclasses	0.90 (0.65-1.24)	0.52	0.93 (0.80-1.08)	0.32
M-LDL-FC	Lipoprotein lipids in subclasses	0.91 (0.67-1.23)	0.54	1.02 (0.92-1.12)	0.76
M-LDL-C	Lipoprotein lipids in subclasses	0.89 (0.64-1.26)	0.52	0.92 (0.79-1.09)	0.33
M-LDL-L	Lipoprotein lipids in subclasses	0.91 (0.65-1.28)	0.6	0.91 (0.78-1.06)	0.23
M-LDL-PL	Lipoprotein lipids in subclasses	0.93 (0.68-1.29)	0.68	0.91 (0.78-1.06)	0.21
M-LDL-CE	Lipoprotein lipids in subclasses	0.93 (0.64-1.33)	0.68	0.90 (0.77-1.05)	0.19
L-LDL-C	Lipoprotein lipids in subclasses	0.95 (0.67-1.34)	0.77	1.00 (0.92-1.08)	0.91
L-LDL-CE	Lipoprotein lipids in subclasses	1.01 (0.73-1.41)	0.94	1.00 (0.92-1.08)	0.9
L-LDL-FC	Lipoprotein lipids in subclasses	1.06 (0.77-1.47)	0.7	1.02 (0.89-1.17)	0.79
L-LDL-L	Lipoprotein lipids in subclasses	1.00 (0.72-1.39)	0.98	1.00 (0.92-1.08)	0.91
L-LDL-PL	Lipoprotein lipids in subclasses	0.99 (0.72-1.38)	0.97	0.93 (0.80-1.08)	0.32
IDL-CE	Lipoprotein lipids in subclasses	1.07 (0.78-1.45)	0.68	1.03 (0.93-1.15)	0.52

Table S12 Sensitivity analyses for two-sample MR analyses.

Exposure	Outcome （sources of RA GWAS）	nSNPs	Weighted median		MR-Egger			
			OR (95% CI)	*P* value	OR (95% CI)	*P* value	Intercept[a]	*P* value
Albumin	Okada	29	1(0.8,1.25)	1	1.03(0.68,1.56)	0.9	-0.009636(0.010049)	0.35
	FinnGen	30	1.15(0.86,1.54)	0.36	1.39(0.93,2.08)	0.12	-0.019124(0.009659)	0.06
GlycA	Okada	44	0.92(0.78,1.07)	0.27	0.86(0.69,1.07)	0.19	0.008852(0.006728)	0.2
	FinnGen	52	0.98(0.78,1.23)	0.85	0.88(0.7,1.12)	0.32	0.013642(0.006783)	0.05
S-HDL-CE	Okada	42	0.89(0.77,1.02)	0.1	1.07(0.83,1.37)	0.6	-0.010446(0.007913)	0.19
	FinnGen	45	1.08(0.9,1.29)	0.42	1.16(0.93,1.45)	0.2	-0.006807(0.006722)	0.32
S-HDL-P	Okada	39	0.89(0.77,1.04)	0.15	1.03(0.77,1.38)	0.84	-0.007465(0.008458)	0.38
	FinnGen	44	0.97(0.79,1.2)	0.79	1.11(0.85,1.44)	0.45	-0.006653(0.007468)	0.38
S-HDL-C	Okada	39	0.89(0.76,1.03)	0.12	0.98(0.74,1.29)	0.86	-0.004776(0.008358)	0.57
	FinnGen	42	0.97(0.79,1.2)	0.8	1.04(0.73,1.48)	0.84	0.002172(0.010247)	0.83
M-LDL-FC	Okada	40	1(0.85,1.17)	1	1.02(0.83,1.25)	0.83	-0.002631(0.00589)	0.66
	FinnGen	49	1.02(0.87,1.2)	0.8	0.99(0.79,1.24)	0.94	0.005161(0.007097)	0.47
S-LDL-FC	Okada	38	1(0.89,1.13)	1	1.1(0.94,1.27)	0.25	-0.007608(0.005354)	0.16
	FinnGen	48	0.98(0.83,1.15)	0.79	0.96(0.78,1.19)	0.73	0.007034(0.007197)	0.33
Omega-3%	Okada	27	1(0.83,1.21)	1	0.99(0.8,1.22)	0.92	0.00558(0.006321)	0.39
	FinnGen	33	1(0.84,1.2)	0.99	1.18(0.97,1.43)	0.1	-0.010529(0.007057)	0.15
L-LDL-C	Okada	40	1(0.86,1.16)	1	1.01(0.85,1.21)	0.89	-0.002551(0.005535)	0.65
	FinnGen	44	1.03(0.87,1.23)	0.71	0.99(0.84,1.18)	0.95	0.001371(0.005867)	0.82
L-LDL-CE	Okada	41	1(0.86,1.16)	1	1.02(0.85,1.21)	0.85	-0.00301(0.005372)	0.58
	FinnGen	45	1.04(0.87,1.23)	0.68	0.99(0.83,1.19)	0.94	0.00189(0.006085)	0.76
L-LDL-FC	Okada	46	1(0.85,1.18)	1	1(0.62,1.62)	1	-0.004723(0.013491)	0.73
	FinnGen	56	1.01(0.85,1.2)	0.9	0.96(0.75,1.23)	0.77	0.006553(0.007482)	0.38
M-LDL-C	Okada	40	0.94(0.81,1.08)	0.39	1.07(0.73,1.57)	0.73	-0.012654(0.011613)	0.28
	FinnGen	45	1.03(0.86,1.24)	0.76	1.01(0.69,1.47)	0.96	-0.004794(0.011929)	0.69
DHA	Okada	35	1.09(0.98,1.22)	0.12	1.23(0.9,1.7)	0.21	-0.015131(0.011867)	0.21
	FinnGen	42	1.09(0.95,1.26)	0.22	1.07(0.88,1.3)	0.49	-0.00181(0.007309)	0.81
Citrate	Okada	32	1(0.83,1.21)	1	0.92(0.7,1.22)	0.56	0.008679(0.007766)	0.27
	FinnGen	34	1.08(0.87,1.34)	0.5	1.23(0.85,1.79)	0.28	-0.012972(0.009774)	0.19
L-LDL-L	Okada	39	1(0.86,1.16)	1	1(0.84,1.2)	0.97	-0.000761(0.005635)	0.89
	FinnGen	43	1.03(0.88,1.21)	0.71	1.01(0.84,1.21)	0.91	-0.000918(0.006272)	0.88
M-LDL-L	Okada	43	0.93(0.8,1.07)	0.31	1.1(0.77,1.59)	0.6	-0.01554(0.010967)	0.16
	FinnGen	48	1.02(0.85,1.21)	0.86	1.01(0.7,1.44)	0.97	-0.006232(0.011311)	0.58
M-LDL-PL	Okada	41	0.91(0.79,1.05)	0.18	1.06(0.75,1.48)	0.75	-0.013195(0.01099)	0.24
	FinnGen	45	1.01(0.86,1.2)	0.87	1.02(0.73,1.43)	0.91	-0.00873(0.011593)	0.46
M-LDL-CE	Okada	44	1(0.85,1.18)	1	1.17(0.75,1.83)	0.48	-0.01749(0.011891)	0.15
	FinnGen	51	0.97(0.81,1.16)	0.73	0.99(0.7,1.4)	0.97	-0.006027(0.010611)	0.57
S-LDL-C	Okada	42	0.98(0.85,1.13)	0.78	1.1(0.79,1.55)	0.57	-0.015815(0.010604)	0.14
	FinnGen	48	1.02(0.86,1.21)	0.82	0.99(0.71,1.37)	0.93	-0.003145(0.010642)	0.77
L-LDL-PL	Okada	38	1(0.86,1.16)	1	1.01(0.85,1.21)	0.9	-0.000774(0.005432)	0.89
	FinnGen	43	1.04(0.88,1.22)	0.64	0.99(0.83,1.18)	0.9	0.003107(0.0059)	0.6
Omega-3	Okada	39	1.01(0.91,1.12)	0.88	1.06(0.92,1.23)	0.43	-0.001293(0.005726)	0.82
	FinnGen	48	1.08(0.96,1.22)	0.21	1.12(0.94,1.32)	0.2	-0.009084(0.007269)	0.22
Polyunsaturated FA	Okada	53	1(0.89,1.13)	1	1.2(0.95,1.51)	0.06	-0.014251(0.007441)	0.06
	FinnGen	57	0.92(0.77,1.09)	0.32	1.07(0.86,1.34)	0.38	-0.005948(0.006789198)	0.38
S-LDL-L	Okada	43	1(0.87,1.15)	1	1.08(0.79,1.48)	0.16	--0.014554(0.010088)	0.16
	FinnGen	48	1.02(0.87,1.2)	0.76	0.98(0.71,1.34)	0.8	-0.002690(0.010642)	0.8
S-HDL-L	Okada	43	0.91(0.78,1.07)	0.25	1(0.43,2.34)	0.84	0.005082(0.024315)	0.84
	FinnGen	45	0.97(0.79,1.18)	0.74	1.06(0.69,1.62)	0.86	-0.002138(0.011968)	0.86
S-HDL-FC	Okada	44	0.9(0.78,1.04)	0.17	0.91(0.67,1.22)	0.87	0.001467(0.00869867)	0.87
	FinnGen	47	0.88(0.72,1.09)	0.25	0.96(0.66,1.41)	0.85	0.002004(0.010239)	0.85
S-LDL-CE	Okada	45	0.97(0.84,1.12)	0.69	1.09(0.77,1.55)	0.14	-0.015726(0.010562)	0.14
	FinnGen	52	0.98(0.82,1.17)	0.81	0.97(0.71,1.34)	0.71	-0.003778(0.010169)	0.71
DHA%	Okada	23	1.2(1.07,1.35)	0	1.18(1.02,1.38)	0.86	-0.0010783(0.005983)	0.86
	FinnGen	29	1.1(0.94,1.27)	0.23	1.13(0.94,1.35)	0.27	-0.008199(0.007273)	0.27
S-LDL-PL	Okada	40	1(0.89,1.13)	1	1.14(0.86,1.5)	0.11	-0.016396(0.010005)	0.11
	FinnGen	46	0.97(0.83,1.14)	0.73	1.01(0.74,1.39)	0.61	-0.005806(0.0112727)	0.61
IDL-CE	Okada	50	1(0.86,1.17)	1	0.94(0.72,1.22)	0.34	0.00685(0.007137)	0.34
	FinnGen	57	1.05(0.89,1.23)	0.57	1.06(0.84,1.36)	0.72	-0.002722(0.007451)	0.72
Valine	Okada	12	0.97(0.76,1.23)	0.79	1.11(0.75,1.66)	0.61	-0.006013(0.011308)	0.61
	FinnGen	17	1.2(0.88,1.66)	0.25	1.26(0.83,1.91)	0.22	-0.014859(0.0116646)	0.22

[a] Okada refer to the study conducted by Okada Y et al. Genetics of rheumatoid arthritis contributes to biology and drug discovery. Nature. 2014;506(7488):376-381. FinnGen refer to the FinnGen biobank analysis round

5 [b] The MR-Egger intercept quantifies the effect of directional pleiotropy. Values that significantly differ from zero provide evidence that the metabolites-associated

Table S13 Associations of metabolic risk score with the risk of RA based on the 37 clinically-validated metabolite set and all 143 measured metabolite set

Categories	MRS on the 37 metabolite set			MRS on the 143 metabolite set		
	Events/total	HR (95% CI) [b]	*P*-value	Events/total	HR (95% CI) [b]	*P*-value
per SD increment	620/93800	1.65(1.54, 1.76)	< 2e-16	620/93800	1.61(1.51-1.72)	< 2e-16
0-50%	171/46900	Ref.		178/46900	Ref.	
50-75%	142/23450	1.38(1.10-1.72)	0.00578	148/23450	1.37(1.09-1.71)	0.005748
75-90%	134/14070	1.96(1.55-2.47)	2.03e-08	120/14070	1.65(1.30-2.11)	3.81e-05
90-100%	173/9379	3.47(2.76-4.36)	< 2e-16	174/9379	3.36(2.67-4.22)	< 2e-16

[a] MRS on the 143 metabolite set included **Albumin, Glycoprotein acetyls, Average diameter for VLDL particles, Saturated fatty acids to total fatty acids percentage, Histidine, and Phospholipids in small LDL**, β-weights for them were -0.279116386, 0.371169131, -0.156626335, 0.017599088, 0.005266765, and -0.073334270, respectively.

[b] Adjusted for age, sex, UK Biobank assessment center, fasting time, education level, smoking status, body mass index, physical activity duration.

Table S14 Associations of the metabolic risk score with the risk of RA with additional adjustments and subgroups.

Category	Adjustment or subgroup	Unit of associations	Hazard ratio	HR (lower)	HR (upper)	*P*-value
Reference model	Age, sex, assmt. center, fasting time, BMI, smoking status, physical activity	SD	2.584	2.2671	2.9458	< 2e-16
Additional adjustments	Age, sex, assmt. center, fasting time, BMI, smoking status, physical activity, prevalent diseases, lipids-lowering drugs and NSAIDS	SD	2.571	2.2578	2.9281	< 2e-16
Additional adjustments	Age, sex, assmt. center, fasting time, BMI, smoking status, physical activity and blood biochemistry measures	SD	2.366	1.9380	2.8874	< 2e-16
Age at blood sampling	<=60	SD	2.871	2.3442	3.516	< 2e-16
Age at blood sampling	>60	SD	2.557	2.17157	3.0098	< 2e-16
Men and women separately	Men	SD	2.910	2.38250	3.5550	< 2e-16
Men and women separately	Women	SD	2.387	2.0034	2.8436	< 2e-16
RF-status	Positive	SD	4.484	3.58754	5.6046	<2e-16
RF-status	Negative	SD	2.310	1.9829	2.691	< 2e-16
Reference model	Age, sex, assmt. center, fasting time, BMI, smoking status, physical activity	90%-100%vs 0-50%	3.522	2.8027	4.4269	< 2e-16
Additional adjustments	Age, sex, assmt. center, fasting time, BMI, smoking status, physical activity, prevalent diseases, lipids-lowering drugs and NSAIDS	90%-100%vs 0-50%	3.529	2.8073	4.4365	< 2e-16
Additional adjustments	Age, sex, assmt. center, fasting time, BMI, smoking status, physical activity and blood biochemistry measures	90%-100%vs 0-50%	2.831	2.1089	3.7996	4.26e-12
Age at blood sampling	<=60	90%-100%vs 0-50%	4.403	3.0852	6.285	3.17e-16
Age at blood sampling	>60	90%-100%vs 0-50%	3.482	2.61848	4.6304	< 2e-16
Men and women separately	Men	90%-100%vs 0-50%	4.645	3.25170	6.6342	< 2e-16
Men and women separately	Women	90%-100%vs 0-50%	2.956	2.2005	3.9710	6.17e-13
RF-status	Positive	90%-100%vs 0-50%	12.86	6.86365	24.0927	1.55e-15
RF-status	Negative	90%-100%vs 0-50%	3.170	2.4614	4.083	1.07e-11

[a] Numerical tabulation of hazard ratios (95% CI) and p-values for results shown in Figure 4

Table S15 Associations of the metabolic risk score with the short-term (2-5 years) and long-term (>5 years) risk of RA.

Categories	Short-term (2-5 years)				Long-term (>5 years)			
	Events/total	HR (95% CI)[a]	*P*-value	*P*-value for trend	Events/total	HR (95% CI)[a]	*P*-value	*P*-value for trend
Per SD increase in MRS		1.4404(1.1557-1.7952)	0.00116			2.2207(1.8313-2.693)	5.02e-16	
0-50%	49/478	Ref.		0.002505	109/46283	Ref.		< 2.2e-16
50-75%	53/371	1.2018(0.8105-1.7820)	0.36035		70/22965	1.0532(0.7766-1.4284)	0.73864	
75-90%	49/271	1.5558(1.0343-2.3402)	0.03385		66/13681	1.4972(1.0912-2.0544)	0.01240	
90-100%	54/334	1.7785(1.1864-2.6661)	0.00531		86/8837	2.6820(1.9745-3.6430)	2.72e-10	

a Adjusted for age, sex, UK Biobank assessment center, fasting time, education level, smoking status, body mass index, physical activity duration.

Table S16 Associations of the metabolic risk score with the risk of RA after excluding incident RA in the first 2 following years.

Categories	Events/total	HR (95% CI)[a]	*P*-value	*P*-value for trend
Per SD increase in MRS		2.3502(2.0289-2.7223)	< 2e-16	
0-50%	158/46761	Ref.		< 2.2e-16
50-75%	123/23336	1.2868(1.0129-1.6347)	0.03890	
75-90%	115/13952	1.8218(1.4206-2.3362)	2.28e-06	
90-100%	140/9171	3.0530(2.3879-3.9032)	< 2e-16	

a Adjusted for age, sex, UK Biobank assessment center, fasting time, education level, smoking status, body mass index, physical activity duration.

Figure S1 A flow diagram of eligible study participants and overall study design

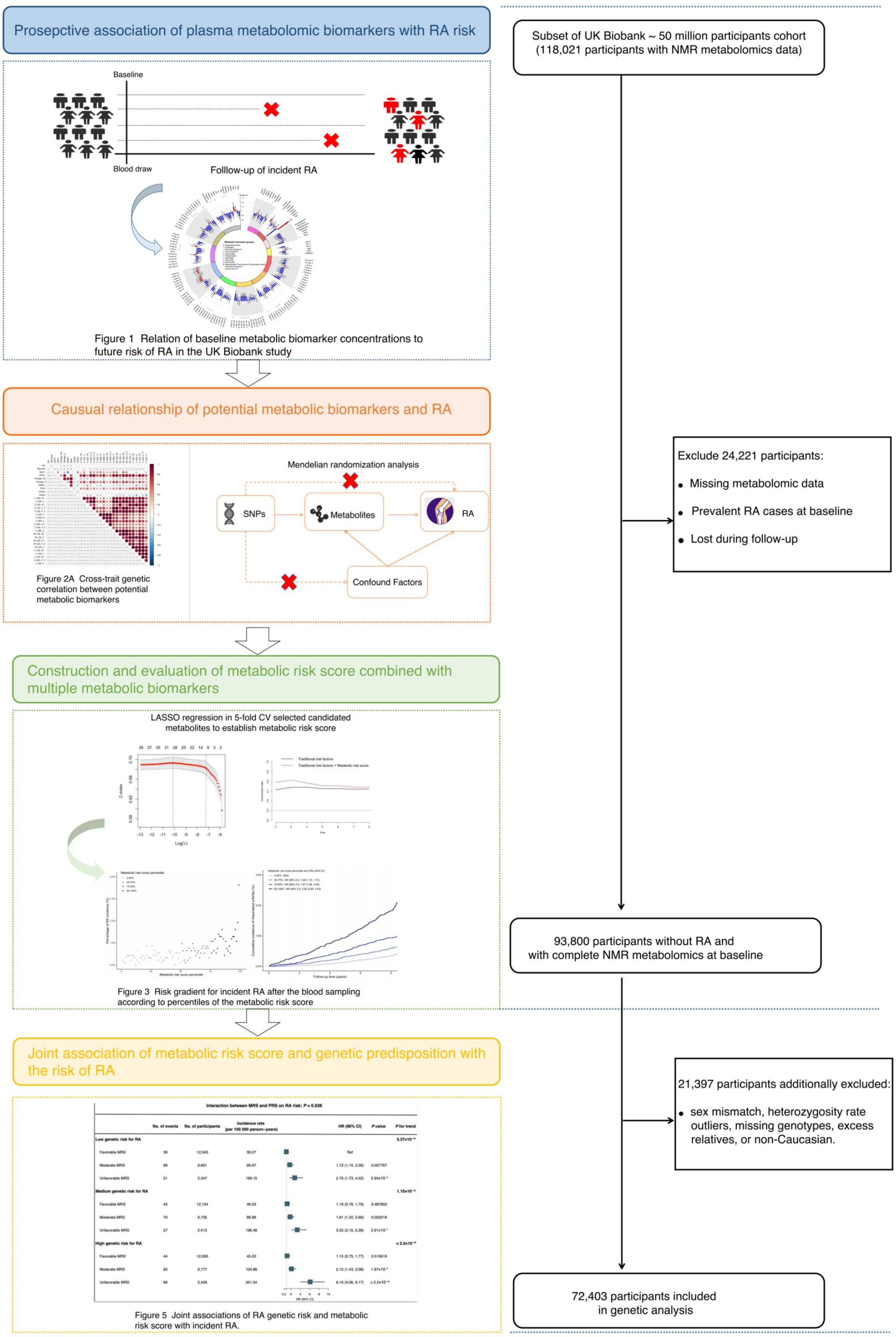

Figure S2 The performance of metabolic risk score in the test dataset and the examination of possible time-varying effects.

(A) **(B)**

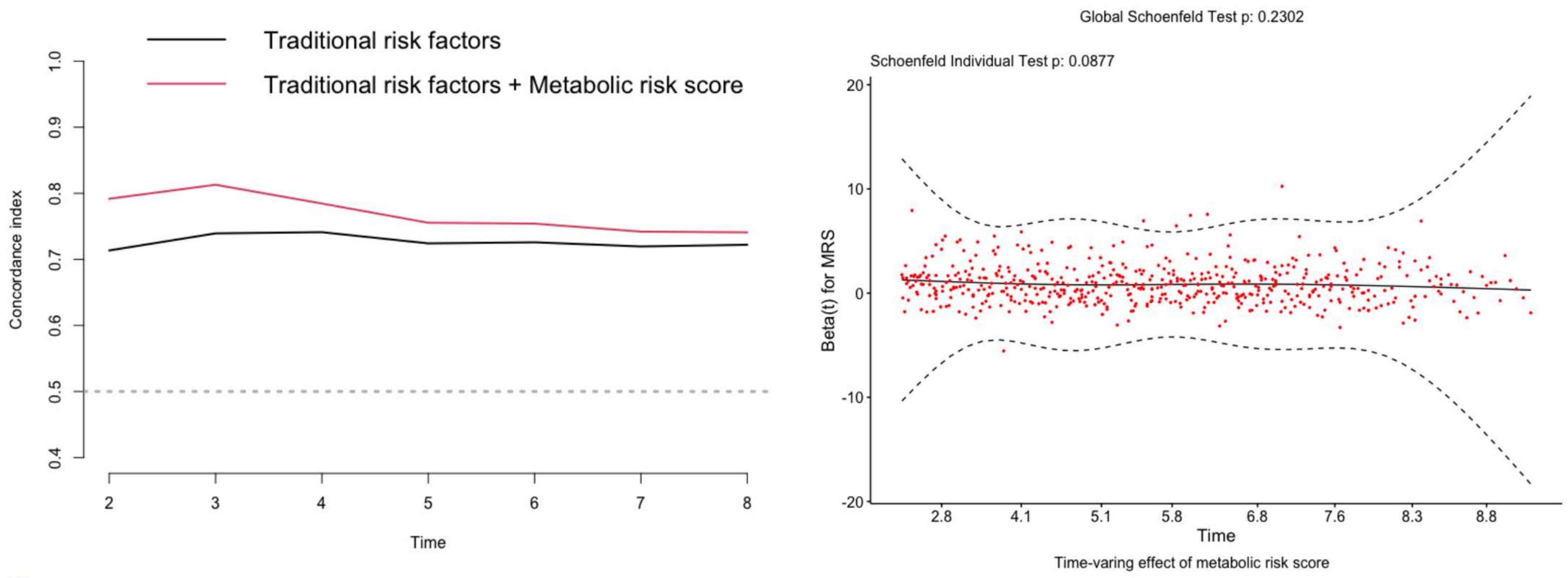

***(A)Concordance index (C-index) comparison between traditional risk factors model (adjusted for age, sex, UK Biobank assessment center, fasting time, education level, smoking status, body mass index, physical activity duration) and combined model (additionally adjusted metabolic risk score)

(B)examination of possible time-varying effects on the metabolic risk score.

Supplemental Figure S3 Spearman correlation coefficients between metabolic risk score and traditional blood biochemistry measures.

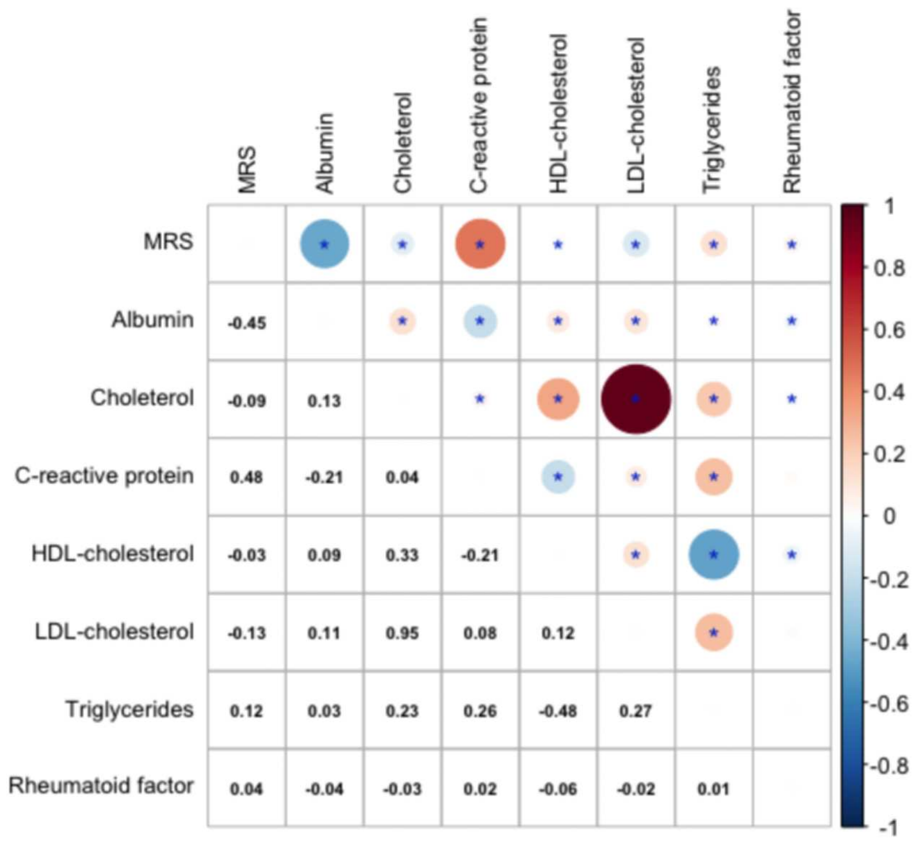

Figure S4 Cumulative incidence of rheumatoid arthritis among subgroups according to gender, age and RF-status.

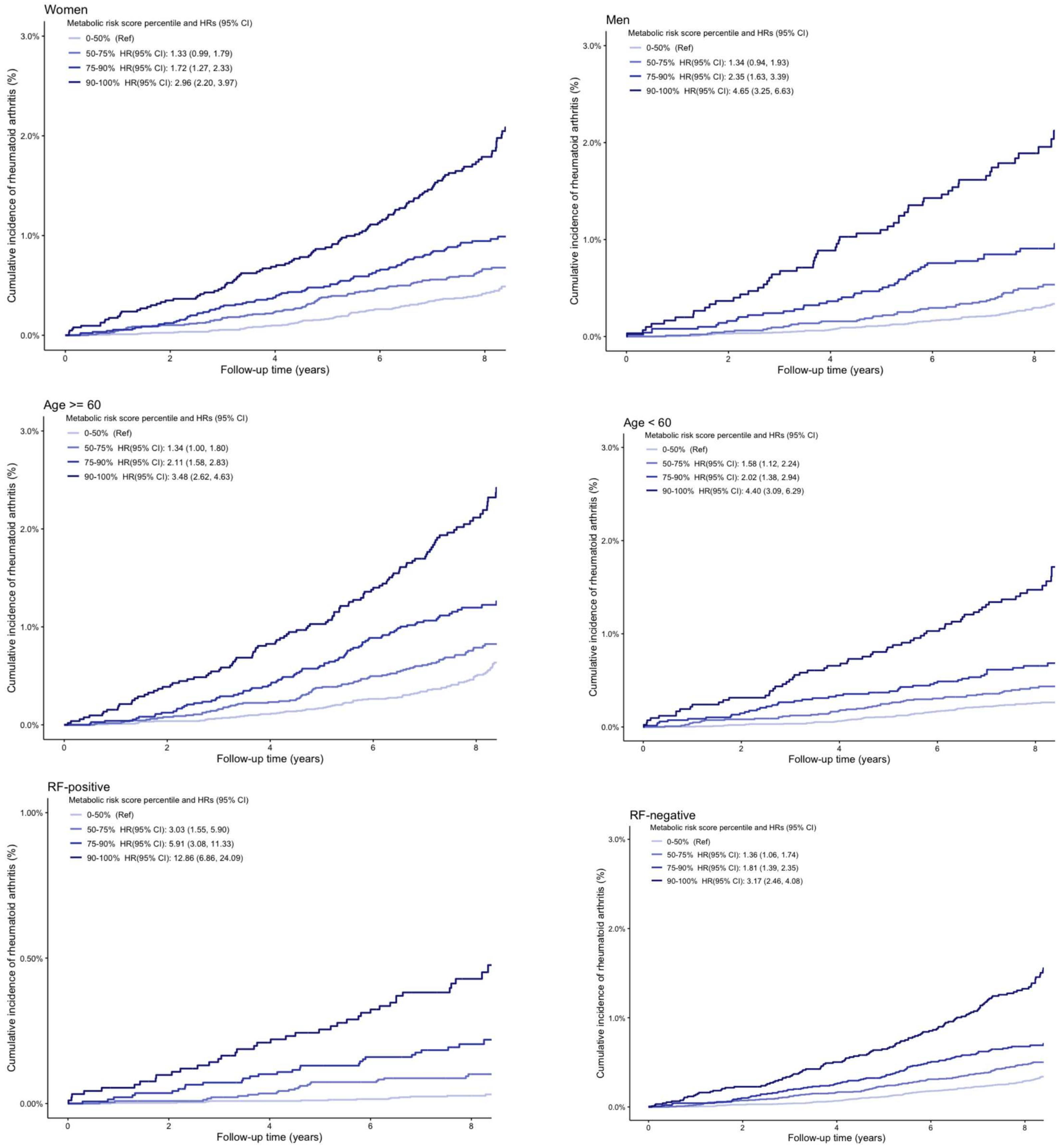

All models were adjusted for age, sex, UK Biobank assessment center, fasting time, education level, smoking status, body mass index, and physical activity duration. For a subgroup of women, additionally adjusted the use of hormone replacement therapy and menopause status.

Figure S5 Joint distribution of metabolic risk score and polygenic risk score

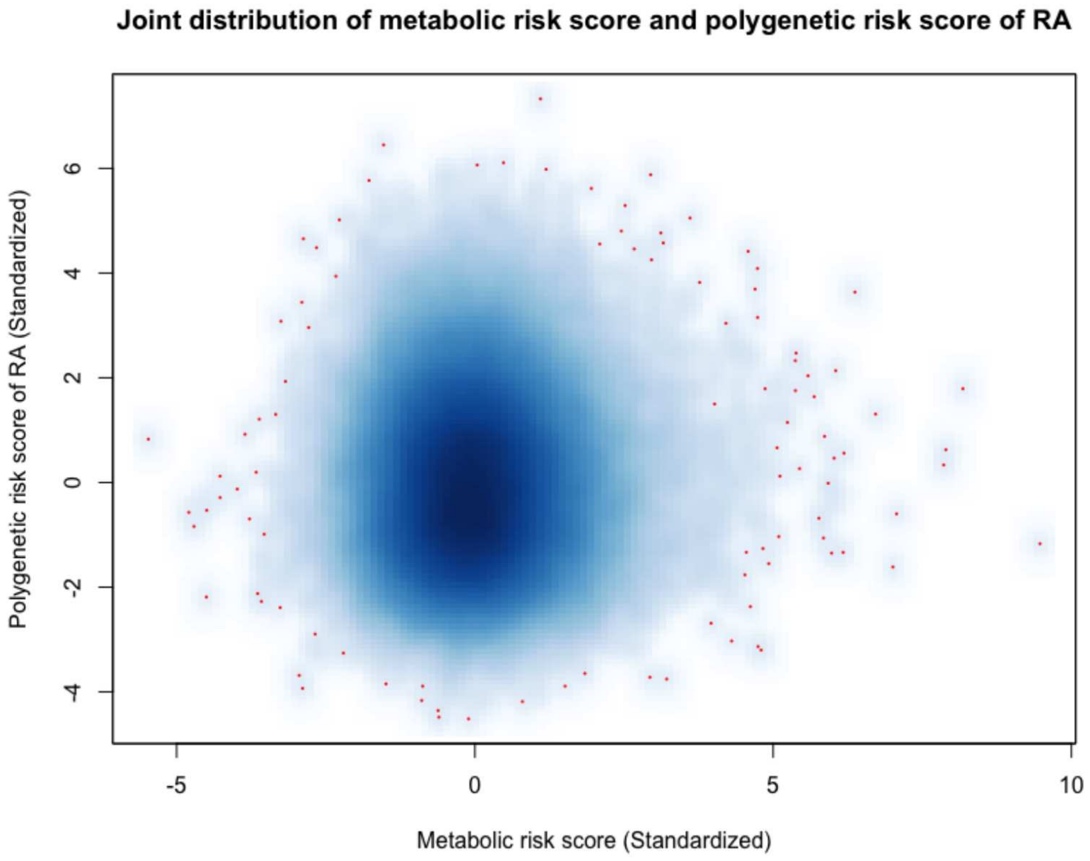

*The blue area represents the density cloud plot of the joint distribution of the metabolic risk score and polygenic risk score. The darker the color represents, the greater density. The red dots represent individuals beyond the 95% range of the joint distribution.
